# Extrapolation Performance of Convolutional Neural Network-Based Combustion Models for Large-Eddy Simulation: Influence of Reynolds Number, Filter Kernel and Filter Size

**DOI:** 10.1007/s10494-025-00643-w

**Published:** 2025-03-24

**Authors:** Geveen Arumapperuma, Nicola Sorace, Matthew Jansen, Oliver Bladek, Ludovico Nista, Shreyans Sakhare, Lukas Berger, Heinz Pitsch, Temistocle Grenga, Antonio Attili

**Affiliations:** 1 School of Engineering, The University of Edinburgh, Edinburgh, EH8 3JL Scotland, UK; 2 Institute for Combustion Technology, RWTH Aachen University, 52056 Aachen, Germany; 3 Faculty of Engineering and Physical Sciences, University of Southampton, Southampton, SO17 1BJ UK

**Keywords:** Deep learning, Convolutional neural networks, Turbulent combustion, Direct numerical simulations, Large-Eddy simulation closure

## Abstract

The extrapolation performance of Convolutional Neural Network (CNN)-based models for Large-Eddy Simulations (LES) has been investigated in the context of turbulent premixed combustion. The study utilises a series of Direct Numerical Simulation (DNS) datasets of turbulent premixed methane/air and hydrogen/air jet flames to train the CNN models. The methane/air flames, which are characterised by increasing Reynolds numbers, are used to model the subgrid-scale flame wrinkling. The hydrogen/air flame, exhibiting complex thermodiffusive instability, is employed to test the ability of the CNN-based combustion models to predict the filtered progress variable source term. This study focuses on the influence of varying training Reynolds numbers, filter sizes, and filter kernels to evaluate the performance of the CNN models to out-of-sample conditions, i.e., not seen during training. The objectives of this study are threefold: (i) analyse the performance of CNN models at different Reynolds numbers compared to the one trained with; (ii) analyse the performance of CNN models at different filter sizes compared to the one trained with; (iii) assess the influence of using different filter kernels (i.e., Gaussian and box filter kernels) between training and testing, to emulate *a posteriori* applications. The results demonstrate that the CNN models show good extrapolation performance when the training Reynolds number is sufficiently high. Vice versa, when CNN models are trained on low-Reynolds-number flame data, their performance degrades as they are applied to flames with progressively higher Reynolds numbers. When these CNN models are tested on datasets with filter sizes not included in the training process, they exhibit sufficient interpolation capabilities, the extrapolation performance is less precise but still satisfactory overall. This indicates that CNN models can be effectively trained using data filtered with a limited range of filter sizes and then successfully applied across a broader spectrum of filter sizes. Furthermore, when CNNs trained on box-filtered data are applied to Gaussian-filtered data, or vice versa, the models perform well for smaller filter sizes. However, as the filter size increases, the accuracy of the predictions diminishes. Interestingly, increasing the quantity of training data does not significantly enhance model performance. Yet, when training data are distributed with greater weighting towards larger filter sizes, the model’s overall performance improves. This suggests that the strategic selection and weighting of training data can lead to more robust generalization across different filter conditions.

**Supplementary Information:**

The online version contains supplementary material available at 10.1007/s10494-025-00643-w.

## Introduction

Recent developments in the field of Machine Learning (ML) have achieved significant breakthroughs in various challenging tasks (Duraisamy et al. 2019), leading to a growing interest in applying ML techniques to turbulence and combustion modelling (Pitsch 2024). Given the increased computational performance of current computing clusters, Large Eddy Simulations (LES) are becoming increasingly common frameworks used to simulate combustion in complex systems of practical relevance, such as gas turbines and aircraft engines. In LES, resolved scales are directly captured by the simulation, while subfilter-scale (SFS) components, which are smaller than the grid resolution, are not directly resolved. Accurate closure models are crucial to account for the interaction of resolved and unresolved scales. Modelling the small-scale interactions between turbulence and chemistry is a challenging task, and the limited predictability of the models has often hindered the use of LES in an industrial context. Given the availability of comprehensive high-fidelity datasets and open-access ML frameworks combined with the availability of computing power through GPUs, data-driven ML modelling has emerged as a promising method to improve the accuracy and efficiency of LES. This opens up new opportunities to improve the predictability and reliability of turbulent combustion simulations, which are crucial for the development and optimisation of the next-generation low-emissions high-efficiency combustion systems. As a result, the use of ML in turbulence and combustion modelling has received significant attention from the combustion community (Brunton et al. 2020; Ihme et al. 2022)

In previous studies (Vollant et al. 2017; Ling et al. 2016; Duraisamy et al. 2015; Maulik and San 2017) Artificial Neural Networks (ANN) were used to model Subgrid-Scale (SGS) terms in turbulent flows for LES and Reynolds Averaged Navier-Stokes Simulations (RANS). However, advances in deep artificial neural networks, such as Convolutional Neural Networks (CNN), are proving to be increasingly useful in modelling turbulent reactive flows (Nista et al. 2023; Ihme et al. 2022), as CNNs are inherently good at learning spatial correlations in the flow field due to the convolution operations performed in each convolutional layer. CNNs are designed to analyse visual representations by extracting features from a dataset (Li et al. 2021). This is well suited for modelling turbulent reacting flows as the SGS wrinkling in a point depends on the spatial structure of the turbulent field in the neighborhood. A study by Nikolaou et al. (2019) showed that CNNs can be successfully used to obtain the unfiltered progress variable field and its variance, an important parameter for an LES with tabulated chemistry. In LES of turbulent premixed flames, the filtered nonlinear reaction rate of the progress variable must be modelled in terms of the resolved fields. Since the turbulent reaction rate is strongly linked to the flame surface area, the task often requires an estimate of the subgrid wrinkling of the flame surface occurring below the resolution of the LES grid. According to previous *a priori* studies, CNNs have been shown to perform remarkably well in approximating the filtered reaction rate (Seltz et al. 2019; Malé et al. 2024) and the subgrid flame wrinkling (Lapeyre et al. 2019).

Despite the remarkable performance of CNNs, *a posteriori* analysis by Lapeyre et al. (2018) highlighted that one of the greatest challenges in using ML models in LES is the ability of the ML model to generalise to new configurations and flow conditions. It has also been observed in many other applications, from speech recognition to medical diagnosis, that ML-based approaches can perform poorly when applied to data that is different from that used for training. Therefore, the models struggle to generalise. Without generalisation capabilities, the supervised data-driven models are limited to physical conditions for which training data is available (Grenga et al. 2023). This issue is particularly relevant for turbulence modelling, as the training data is usually obtained using high-fidelity Direct Numerical Simulations (DNS). However, due to the high computational cost of performing DNSs, producing high-quality data under conditions relevant to real-world applications such as gas turbines and engines is computationally prohibitive. Therefore, training data for ML models can only be obtained at relatively low Reynolds numbers compared to real applications. In the context of turbulent combustion modelling, the disparity in the Reynolds number, flow configuration, and geometrical complexity are arguably the most notable differences between industrial scale devices and configurations amenable for DNS or experiments, that are suitable for the production of training data. Without generalisation capabilities, supervised data-driven models are limited to physical conditions for which DNS or experimental data are available. For these reasons, it is crucial to investigate how well CNN-based, and more general, ML-based models, perform when applied to conditions that are different from those of the training data.

Another aspect that deserves further analysis is that the filter kernel definition and width in an actual LES are not explicitly known. This is due to a number of reasons, including the implicit filtering effect of the LES grid and the artificial dissipation introduced by the numerical scheme. However, a specific filter kernel definition and width is usually employed when preprocessing the DNS data used to train the models (Nista et al. 2024). Therefore, it is important to understand how CNN-based models perform when the filter kernel and size used to train and test the models are different.

In the present work, an *a priori* study based on a CNN (Lapeyre et al. 2019) is first applied to model the subgrid flame wrinkling in a series of four DNSs of turbulent premixed methane/air jet flames with increasing Reynolds number (Luca et al. 2019; Attili et al. 2021). In addition, a DNS of a turbulent premixed hydrogen/air jet flame (Berger et al. 2022) is employed to model the filtered progress variable source term. The purpose of using the hydrogen/air dataset is to test the CNN models in predicting a field with a different underlying physics, such as the filtered progress variable source term in the thermodiffusively unstable hydrogen/air flame. In this flame, the reaction rate (i.e., the progress variable source term) is strongly inhomogeneous along the flame front. The heavily wrinkled flame front contains regions with high reaction rates followed by local extinction. Therefore, it is of interest to study how the CNN models learn these geometric patterns and, more importantly, how well these models can be generalised when applied to data that has not been used to train the models.

The goal of using an *a priori* analysis in this study is to train and test a broad range of different models over a large set of test cases. This broad exploration helps to identify key features of the CNN models that can be crucial for accurate combustion modelling. In addition, this approach enables a systematic analysis of the performance of the CNN models to identify any challenges and limitations in using CNNs for combustion modelling. Addressing these challenges *a priori* lays the foundation for more robust *a-posteriori* validation in future studies. However, *a posteriori* analysis of CNN models is challenging due to the difficulty of integrating Python-based ML models with Computational Fluid Dynamics (CFD) codes typically written in C++ or Fortran. Bridging these languages requires resolving differences in design and runtime environments. Additionally, deep learning models trained on structured data often need interpolation and preprocessing to handle irregular grids used in unstructured solvers. This leads to additional computational effort and possible interpolation errors (Heaney et al. 2024). A study by Nista et al. (2025) has also highlighted some of the challenges that arise when coupling ML models with a massively parallelised CFD solver. In a parallel runtime environment, the transfer of data between the ML model and the CFD solver can be computationally expensive. Therefore, their study used a heterogeneous cluster architecture, where the CFD solver is executed on the central processing units (CPU), while the ML inference is performed on the corresponding graphical processing unit (GPU) available to the same CPU node. This type of hybrid architecture has been shown to reduce the data transfer bottlenecks while having a negligible impact on the parallel scalability of the solver. Even though a different ML architecture was used in their study, the challenges encountered are common to all deep learning models. Carefully addressing these challenges is still a subject of ongoing research that requires a lot of time and effort, which is crucial for creating a robust and efficient coupling between the CNN and the CFD code.

For the aforementioned reasons, the objectives of this study are threefold:

i. Train four CNN models using the data from each of the four methane/air flames and test each of them on all four flames to evaluate the performance of ML-based models when applied to data different from those used for training. Filter kernel and size are kept constant for training and testing data to isolate the generalisation of the models to different Reynolds numbers. Particular effort is given to the assessment of the performance of models trained with low-Reynolds number cases and applied to high-Reynolds number flames to investigate the ability to extrapolate toward conditions of industrially relevant devices.
ii. Assess the generalisation of the CNN models to filter size. Filter the methane/air and hydrogen/air DNS datasets with a range of filter sizes with either a Gaussian or a box filter kernel; then train CNN models with a combination of filter sizes and test them using data with filter sizes that were not used to train the models. The aim is to study the performance of the CNN model when applied to different filter sizes that were not used to train the models.
iii. Assess the generalisation of the CNN models to filter kernel. Filter the DNS data with either a Gaussian or a box filter kernel while keeping the filter size constant; then train CNN models with data filtered with either filter type and apply the models to data filtered with a kernel type that was not used to train the model. The aim is to study the extrapolation performance of the CNN model when applied to different filter types that were not used to train the models.

The last two objectives emulate the application of using trained models on data for which the implicit filter operation is unknown.

## DNS Datasets Description

### Methane/Air Premixed Flames

A series of turbulent premixed jet flames with increasing Reynolds number (Re), and approximately constant Karlovitz number (Ka), has been employed (Luca et al. 2019; Attili et al. 2021) for training and *a priori* testing. A slot turbulent premixed jet flame with an equivalence ratio $\Phi$ = 0.7, a temperature of 800 K, and a pressure of 4 atm, surrounded by a coflow of burnt gases, is considered. A summary of all relevant flow parameters can be found in Table 1. Based on one-dimensional simulations of a freely propagating flame, the laminar flame speed is $S_L = 1 \,\mathrm {ms^{-1}}$ and the thermal thickness is $\delta _L = 110 \,\mu m$ for all four configurations.

**Table 1:** Simulation parameters for the methane/air turbulent flames, evaluated in the fully turbulent region. H is the slot width, $\mathrm {U_{jet}}$ is the bulk velocity of the jet, $u^\prime$ is the turbulence intensity, *l* is the integral length scale, $\eta$ is the Kolmogorov length scale, $\mathrm {Re_\lambda }$ is the Reynolds number based on the Taylor microscale evaluated in the unburnt jet, and $N_x$, $N_y$, $N_z$ are the number of grid points in the three spatial directions

Case	R1-K1	R2-K1	R3-K1	R4-K1
H (mm)	0.6	1.2	2.4	4.8
$\mathrm {U_{jet}}$ (m/s)	100	100	100	100
Re	2800	5600	11200	22400
$u^\prime$ (m/s)	14.3	10.1	9.9	11.7
*l* (mm)	0.54	0.54	0.67	1.1
$\eta$ ($\mathrm {\mu m}$)	18	23	25	25
$u^\prime /S_L$	14.2	10.0	9.8	11.6
$l/\delta _L$	4.8	4.8	5.9	9.5
Ka	39	23	21	21
$\mathrm {Re_\lambda }$	49	39	40	50
$N_x$	720	1440	2880	5760
$N_y$	480	960	1920	3840
$N_z$	256	256	512	1024

Since the four DNS cases have different Reynolds numbers, the case name ranges from R1 to R4 which stands for a low to high Reynolds number. In addition, all four case names contain K1, which indicates a constant Karlovitz number. The size of the computational domain is 24 H in the streamwise (*x*), 16 H in the crosswise (*y*), and 4.3H in the spanwise (*z*) direction (8.5H for R1-K1). A fully structured mesh is employed with a grid size of $dx = 20\, \mu m$ resulting in an 88 million cell mesh for R1-K1, 350 million for R2-K1, 2.8 billion for R3-K1, and 22 billion for R4-K1. The resolution is such that $\delta _L /dx \approx 6$ and $dx/\eta <2$ at all locations.

**Fig. 1:**
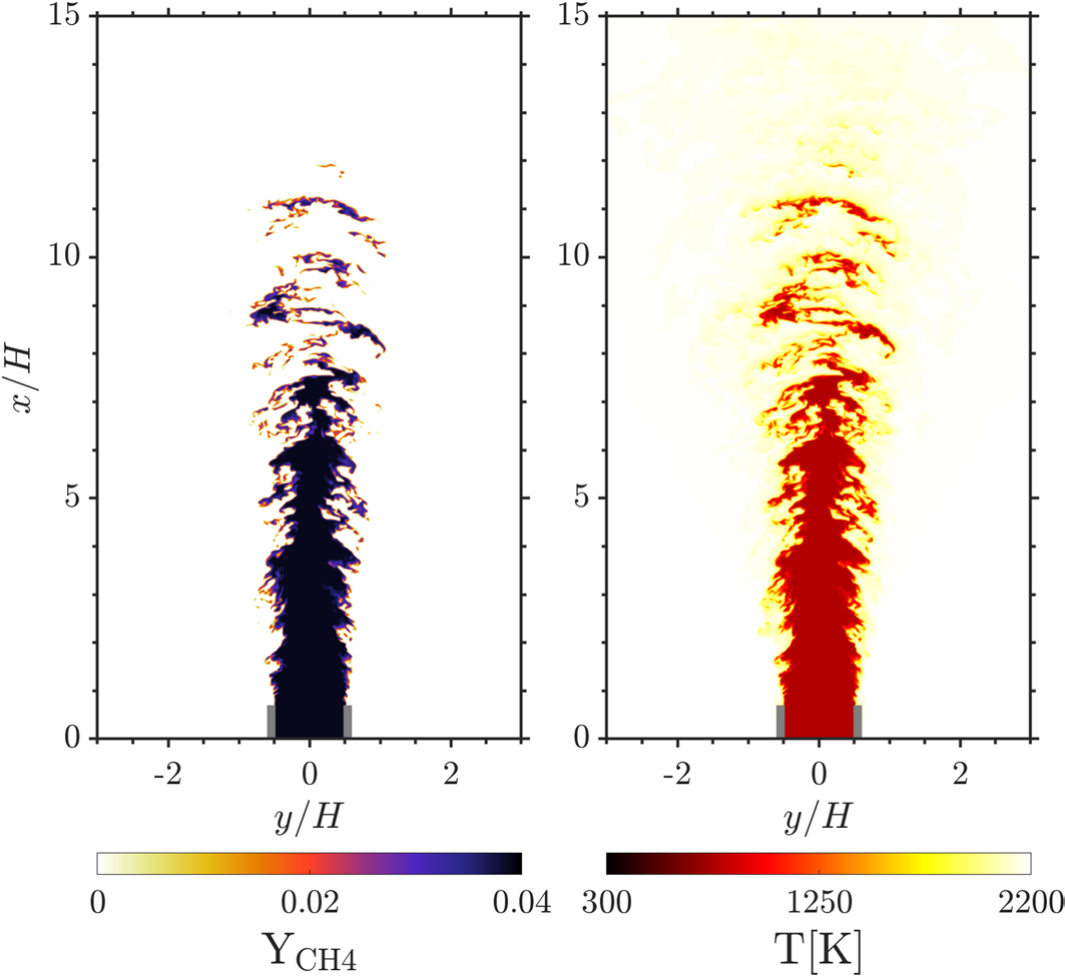
Instantaneous snapshots of the fuel mass fraction (left) and the temperature (right) contour of the turbulent methane/air flame for the configuration R4-K1 (Table 1). The grey lines indicate the slot walls

The domain is periodic in *z*, open boundary conditions are prescribed at the outlet in *x* and slip conditions are imposed at the boundaries in *y*. The inlet conditions for the velocity field are obtained from four auxiliary fully developed turbulent channel flow DNS. The instantaneous realisations of the channel flow are sampled in time to create the inlet velocity profile. The reactive, unsteady Navier Stokes equations are solved in the low Mach number limit (Tomboulides et al. 1997). All transport properties are computed with a mixture-average approach (Attili et al. 2016) and a skeletal methane mechanism with 16 species and 72 reactions (Luca et al. 2018) is employed. Figure 1 shows the fuel mass fraction ($\mathrm {Y_{CH_4}}$) and the temperature of the R4-K1 configuration.

### Hydrogen/Air Premixed Flame

A large-scale DNS of a three-dimensional turbulent premixed lean hydrogen/air flame is also employed in this study (Berger et al. 2022) for training and *a priori* testing. It is a slot burner configuration with an equivalence ratio of $\Phi$ = 0.4, a temperature of 298K, and a pressure of 1 bar, surrounded by a coflow of burned gasses. This DNS features strong thermodiffusive instabilities due to the low Lewis number of hydrogen. A summary of all relevant flow parameters of the flame are reported in Table 2.

**Table 2:** Simulation parameters for the hydrogen/air turbulent flame, evaluated in the fully turbulent region

Case	$\mathrm {H_2/Air}$ flame
H (mm)	8
$\mathrm {U_{jet}}$ (m/s)	24
$\mathrm {U_{coflow}}$ (m/s)	3.6
$S_L$ (m/s)	0.17
$\mathrm {\delta _L (\mu m)}$	714
$\eta$ ($\mathrm {\mu m}$)	180
Re	11000
Ka	20
$N_x$	1792
$N_y$	1024
$N_z$	512

**Fig. 2:**
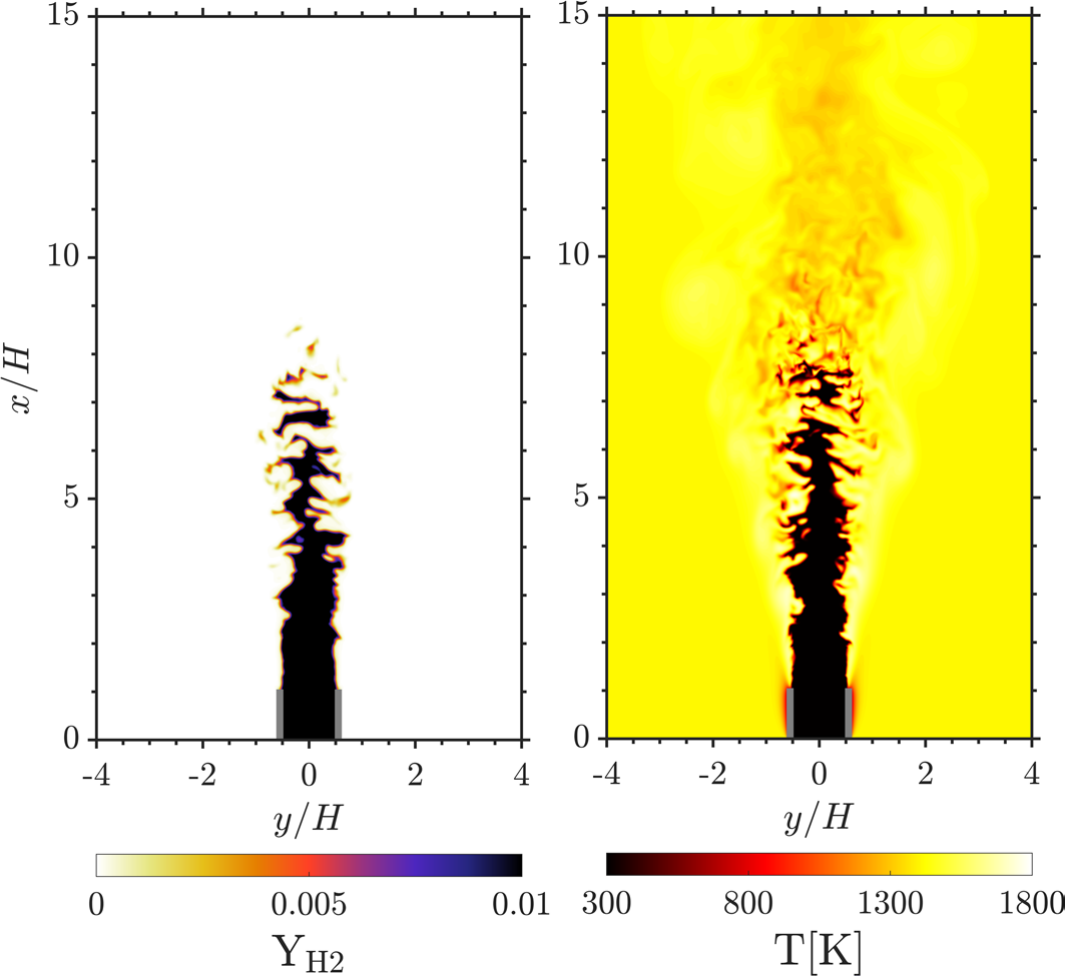
Instantaneous snapshots of the fuel mass fraction (left) and the temperature (right) contour of the turbulent hydrogen/air flame. The grey lines indicate the slot walls

The size of the computational domain is 15 H in the streamwise (*x*), 12.5H in the crosswise (*y*), and 4.6H in the spanwise (*z*) direction. A fully structured mesh is employed with a grid size of $dx = 70\, \mu m$ resulting in a $\approx$ 940 million cell mesh. The resolution is chosen so that $\delta _L /dx \approx 10$ and $dx/\eta <2$ at all locations. The domain is periodic in *z*, open boundary conditions are prescribed at the outlet in *x* and slip conditions are imposed at the boundaries in *y*. The inlet conditions for the velocity field are obtained from a fully developed turbulent channel flow DNS. The instantaneous realisations of the channel flow are sampled in time to create the inlet velocity profile. The chemical reactions are modelled by the mechanism of Burke et al. (2011), which contains 9 species and 46 reactions. Figure 2 shows the fuel mass fraction ($\mathrm {Y_{H_2}}$) and the temperature of the turbulent premixed hydrogen/air flame.

## Mathematical Formulation

### Modelling Flame Surface Density

In the context of turbulent premixed combustion modelling, the combustion progress is described by a scalar field, called progress variable, that is strained and stretched by the combined effects of the turbulent velocity field, diffusion and chemical reactions. The progress variable describes the combustion progress from unburnt, when C = 0, to fully burnt, when C = 1. In the context of the methane/air premixed flames described in Sect. 2.1, the progress variable is defined in terms of the mass fraction of $\mathrm {CH_4}$:

1 $$\begin{aligned} C = 1 - \frac{\mathrm {Y_{CH_4} - Y_{CH_4,min}}}{\mathrm {Y_{CH_4,max} - Y_{CH_4,min}}} \end{aligned}$$

The progress variable is normalised between the minimum and maximum values of the $\mathrm {CH_4}$ mass fraction from the DNS such that for a fully burnt condition C = 1 and for a fully unburnt condition C = 0.

In the context of LES, a modelled density-weighted (Favre) filtered progress variable equation in terms of $\widetilde{C}$ is defined as:

2 $$\begin{aligned} \frac{\partial \bar{\rho } \widetilde{C}}{\partial t}+ \nabla \cdot \left( \bar{\rho } \tilde{u} \widetilde{C}\right) + \nabla \cdot \left( \bar{\rho } \widetilde{u C} -\bar{\rho } \tilde{u} \widetilde{C} \right) =\nabla \cdot \, \left( \bar{\rho } \widetilde{D} \nabla \widetilde{C}\right) + \overline{\dot{\omega }}, \end{aligned}$$

where $\bar{\rho }$ is the filtered density, $\tilde{u}$ is the Favre filtered velocity, the first term on the right-hand side is the filtered diffusive fluxes, where $\widetilde{D}$ is the molecular diffusivity. The unresolved diffusive fluxes are usually neglected, therefore, they are not included in the equation, $\overline{\dot{\omega }}$ is the filtered reaction source term. This equation contains unclosed terms that cannot be expressed as a function of the known variables and require modelling. On the right-hand side of Eq. 2, the filtered diffusion and the source term can be incorporated into a single term as Knikker et al. (2004):

3 $$\begin{aligned} \nabla \cdot \, \left( \bar{\rho } \widetilde{D} \nabla \widetilde{C}\right) + \overline{\dot{\omega }} = \overline{\rho S_d |\nabla C|}, \end{aligned}$$

where $S_d$ is the displacement speed of the *C*-isosurface, often assumed to be equal to the laminar flame speed $S_L$. The right-hand side of Eq. 3 can be modelled as Boger et al. (1998):

4 $$\begin{aligned} \overline{\rho S_d |\nabla C|} \approx \rho _{u} S_L \overline{\Sigma }, \end{aligned}$$

where $\rho _{u}$ is the unburnt gas density. $\overline{\Sigma } = |\overline{\nabla C}|$ is the flame surface density, which, in general, cannot be obtained from the resolved field. To overcome this, the model is often formulated in terms of the wrinkling factor:

5 $$\begin{aligned} \Xi = \frac{\overline{\Sigma }}{|\nabla \overline{C}|} = \frac{|\overline{\nabla C}|}{|\nabla \overline{C}|}, \end{aligned}$$

which describes the ratio between the resolved (filtered) flame surface and its unfiltered value. It is worth noting that, although the problem is formulated here in a specific form, the task of modelling the subfilter wrinkling and the flame area is also relevant to other modelling strategies based on different approaches.

In summary, the modelling task requires to compute the flame surface density $\overline{\Sigma } = |\overline{\nabla C}|$ from the filtered quantity $\overline{C}$. This will be computed using a CNN model. The model will be trained to learn the relation between an input field, in this case, the filtered progress variable $\overline{C}$ and the desired output $\overline{\Sigma }$. The data for training the CNN models is obtained from the methane/air DNS dataset. In addition, there are several approaches in the literature for modelling $\overline{\Sigma }$ or similar tasks that are either algebraic or based on the solution of differential equations (Charlette et al. 2002a, b; Wang et al. 2011; Pitsch 2006. Among these modelling approaches, a well-known and largely applied algebraic model proposed by Charlette et al. (2002a) will be employed to model $\overline{\Sigma }$. The results of the CNN models will then be compared to the algebraic model to evaluate the performance of the CNN models. The algebraic model that will be referred to as the Charlette model, is formulated as follows:

6 $$\begin{aligned} \overline{\Sigma }=\left( 1+\min \left[ \frac{\Delta }{\delta _L}-1, \Gamma _{\Delta }\left( \frac{\Delta }{\delta _L}, \frac{u_{\Delta }^{\prime }}{S_L}, R e_{\Delta }\right) \frac{u_{\Delta }^{\prime }}{S_L}\right] \right) ^\beta |\nabla \bar{c}| \end{aligned}$$

where $\Delta$ is the filter width, $u_{\Delta }^{\prime }$ is the subgrid turbulent velocity up to $\Delta$, $Re_{\Delta } = u_{\Delta }^{\prime } \Delta /\nu$ is the SGS Reynolds number, and $\beta$ is a model constant. In this study, a parameter value of $\beta =0.5$ is employed, as the same value was employed in previous studies (Lapeyre et al. 2019; Charlette et al. 2002a) as well. The performance of the Charlette model with different $\beta$ values was evaluated and a value of 0.5 provides the best results over a wide range of filter sizes as shown in the supplementary material. $\Gamma _{\Delta }$ is a function that accounts for the straining effects on the flame structure due to turbulent scales smaller than $\Delta$ Meneveau and Poinsot (1991). Details regarding the formulation of $\Gamma _{\Delta }$ can be found in Charlette et al. (2002a).

### Modelling the Progress Variable Source Term in Premixed Hydrogen/Air Flames

In lean hydrogen flames, there is a significant disparity between the molecular and thermal diffusion, which is characterised by the low Lewis number of hydrogen. This leads to strong differential diffusion effects within the flame front. The large disparity between the thermal and hydrogen mass flux leads to an amplification of the small perturbations in the flame front resulting in thermodiffusive instabilities, that strongly affects the flame structure and dynamics, as shown by Berger et al. (2022a, 2022b). In addition, Aspden et al. (2011, 2019) and Berger et al. (2022) have demonstrated that turbulence enhances the thermodiffusive effects, except in the case of very large Karlovitz numbers (Aspden et al. 2011, 2019). It was found that turbulence and instability interact synergistically, resulting in the thermodiffusive effects being even stronger in turbulent flames than in a laminar counterpart.

Similar to the mathematical formulation of the methane flames in Sect. 3.1, the progress variable is used to describe the progress of premixed hydrogen combustion. For the hydrogen investigations, the progress variable is defined in terms of the mass fraction of $\mathrm {H_2O}$:

7 $$\begin{aligned} C = \frac{\mathrm {Y_{H_20} - Y_{H_2O,min}}}{\mathrm {Y_{H_2O,max} - Y_{H_2O,min}}} \end{aligned}$$

The density-weighted filtered progress variable transport equation is identical to Eq. 2 in Sect. 3.1.

Since the progress variable in this context is defined in terms of $\mathrm {H_2O}$, the unclosed filtered reaction source term in Eq. 2 will be referred to as $\overline{\dot{\omega }}_{\mathrm {H_2O}}$, which in general cannot be obtained from the resolved field. Therefore, in an LES context, $\overline{\dot{\omega }}_{\mathrm {H_2O}}$  must be modelled.

Due to the aforementioned characteristics of lean premixed hydrogen/air flames, conventional combustion models generally perform poorly and often fail to capture the effects of thermodiffusive instabilities (Berger et al. 2022). Unlike methane, the reaction rate of hydrogen flames cannot be parameterised by the progress variable only (Berger et al. 2022). A second variable, such as an additional progress variable (Remiddi et al. 2024; Bastiaans et al. 2007), a mixture fraction (Regele et al. 2013; Schlup and Blanquart 2019; Berger et al. 2022), or the flame curvature (Böttler et al. 2023), is therefore required to parameterise the local reaction rate. In this work, a CNN is trained to learn the relationship between a single input field, in this case, the filtered progress variable $\overline{C}$ and to predict the desired output $\overline{\dot{\omega }}_{\mathrm {H_2O}}$. The data for training the CNN models is obtained from the hydrogen/air DNS dataset. In addition, $\overline{\dot{\omega }}_{\mathrm {H_2O}}$ is also obtained from a flamelet table containing a collection of one-dimensional unstretched laminar flames with different equivalence ratios to account for the local variations of mixture fraction due to thermodiffusive instabilities. This is a model that works well in laminar premixed flames (Schlup and Blanquart 2019). For turbulent premixed flames this model has been extended by assuming a subfilter Probability Density Function (PDF), so additionally the variance of progress variable is also needed (Berger et al. 2024). Therefore, the table is parameterised using progress variable, progress variable variance and mixture fraction (Berger et al. 2024, 2022). The predictions of the CNN models are then compared with the results from the flamelet table to evaluate the performance of the CNN models.

## CNN Architecture and Training

### Neural Network Architecture

In this study, the objective is to use the CNN model to map the 3D input field $\overline{C}$ at every mesh point to a corresponding $\overline{\Sigma }$ or $\overline{\dot{\omega }}_{\mathrm {H_2O}}$  value, resulting in an output field with the same dimensions as the input field. A CNN-based architecture is better suited for this task as it can learn to map the entire field resulting in a direct field-to-field map rather than a collection of individual node values. Conceptually, this task is very similar to an ML task known as image segmentation, where an input image is analysed and each pixel is classified to identify different patterns in the image. However, a typical CNN classifies an image with a single label, i.e., it does not classify local information. For image segmentation, every pixel in the image must be classified. To improve the localisation performance, Ronneberger et al. (2015) modified and extended a fully convolutional neural network (Long et al. 2015) and developed the so-called U-Net architecture which can be trained with few training images and yields more precise segmentation.

The U-Net developed in Ronneberger et al. (2015) takes a 2D image as the input and produces a 2D output and is optimised for 2D biomedical image segmentation. Lapeyre et al. (2019) adapted the U-Net to handle 3D input/output data and model the subgrid-scale flame wrinkling in premixed methane/air flames. Their study showed remarkable success in predicting the subgrid-scale flame wrinkling. In this study, the U-Net used in Lapeyre et al. (2019) is employed to study the extrapolation performance of the model when applied to data that was not used to train the model.

**Fig. 3:**
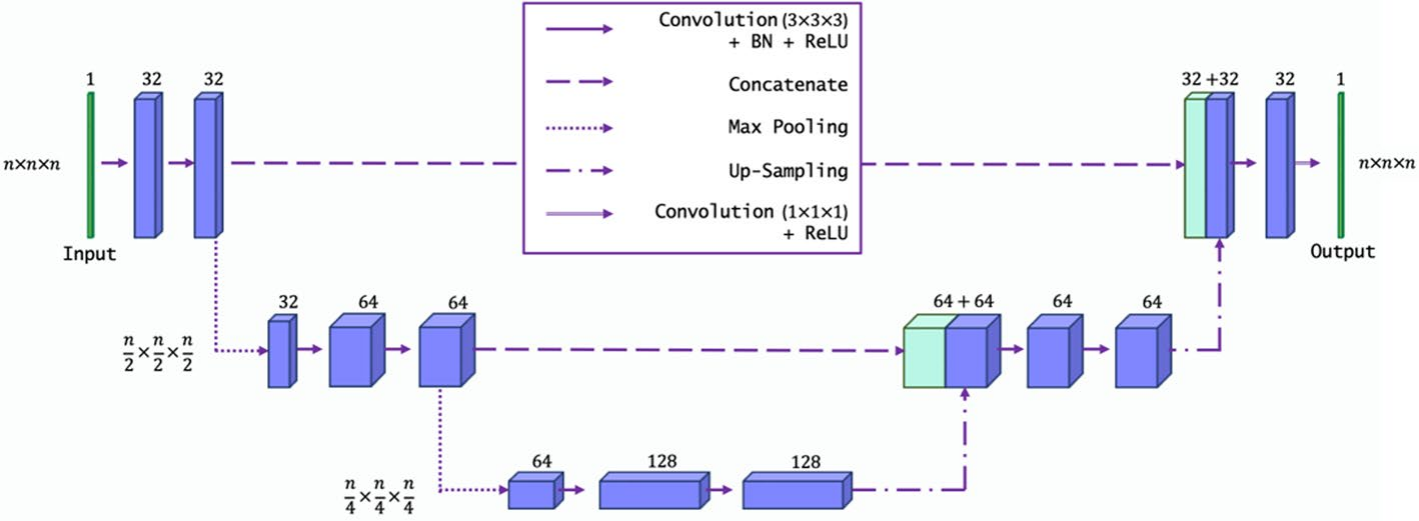
3D U-Net architecture. The CNN is constructed using a total of 13 layers and the numbers above each layer represent the number of filters used for each convolutional layer

Figure 3 shows the U-Net architecture used in this study. It is a fully convolutional neural network consisting of 13 layers with standard downsampling and upsampling operators. Each downsampling step consists of a padded 3D convolution with a $3\times 3\times 3$ kernel, followed by a Batch Normalisation (BN) and a Rectified Linear Unit (ReLu) activation. The downsampling steps include $2\times 2\times 2$ max pooling operations and the number of feature channels is doubled. The upsampling steps have a very similar structure to the downsampling steps. They include 3D transposed convolutions instead of 3D convolutions followed by $2\times 2\times 2$ upsampling operations to recover the original dimension of the input field. In addition, the final layer contains a $1\times 1\times 1$ convolution followed by a ReLu activation to prevent the U-Net from predicting negative values. The network contains a total of 1.5 million trainable parameters. From now on, the U-Net is simply referred to as the CNN.

### Training the CNN

The training data for the CNN are obtained from the two DNS datasets described in Sects. 2.1 and 2.2. To train and test the CNN models, the data is classified into three categories:

i. *The training data* Contains input fields and corresponding ground-truth fields. Therefore, supervised learning is performed to train the CNN.
ii. *The validation data* A different set of data samples from the same dataset as the training data. This is used to evaluate the performance of the CNN model after every epoch during the training. The validation data is used in the validation step during training which acts as an intermediary step between the training and testing stages. Even though the training data is used to adjust the model parameters, which aims to minimise errors and improve the prediction accuracy, evaluating the model performance solely based on the training data can lead to overfitting. Therefore, validation data helps to mitigate this by providing an independent set of data samples, that allow an assessment of the model’s generalisation capability and fine-tuning of the model parameters to improve the robustness of the model.
iii. *The testing data* Can be data from the same dataset used for training or from a completely different dataset with different flow conditions, different filter sizes and filter kernels. In either case, it is data that is never seen by the models during training. The testing data is used after the training process of the models is completed to assess the performance of the trained CNN models.

In this study, the training data is obtained by filtering the DNS data with either a box or a Gaussian filter kernel to emulate LES-like fields. The mathematical representation of the 3D box filter kernel is as follows:

8 $$\begin{aligned} \overline{\xi (i,j,k)} = \frac{1}{(\Delta +1)^3} \sum _{m=-\Delta /2}^{\Delta /2} \sum _{n=-\Delta /2}^{\Delta /2} \sum _{p=-\Delta /2}^{\Delta /2} \xi (i+m, j+n, k+p), \end{aligned}$$

where $\xi$ is the filtered DNS field and $\Delta$ is the filter width. The box filter kernel adopted averages all cell values in the DNS within the given filter width. In addition, a Gaussian filter kernel is also used. The mathematical representation of the 3D Gaussian filter kernel is as follows:

9 $$\begin{aligned} \overline{\xi (i,j,k)} = \frac{1}{ \left( {\sigma \sqrt{2\pi }}\right) ^3} \sum _{m=-\Delta /2}^{\Delta /2} \sum _{n=-\Delta /2}^{\Delta /2} \sum _{p=-\Delta /2}^{\Delta /2} e^{-\frac{m^2 + n^2 + p^2}{2\sigma ^2}} \xi (i+m, j+n, k+p), \end{aligned}$$

where $\sigma = (\Delta -1)/2$ is the standard deviation. The Gaussian kernel averages the DNS field over the specified filter width, giving greater weight to values closer to the centre of the transfer function.

**Fig. 4:**
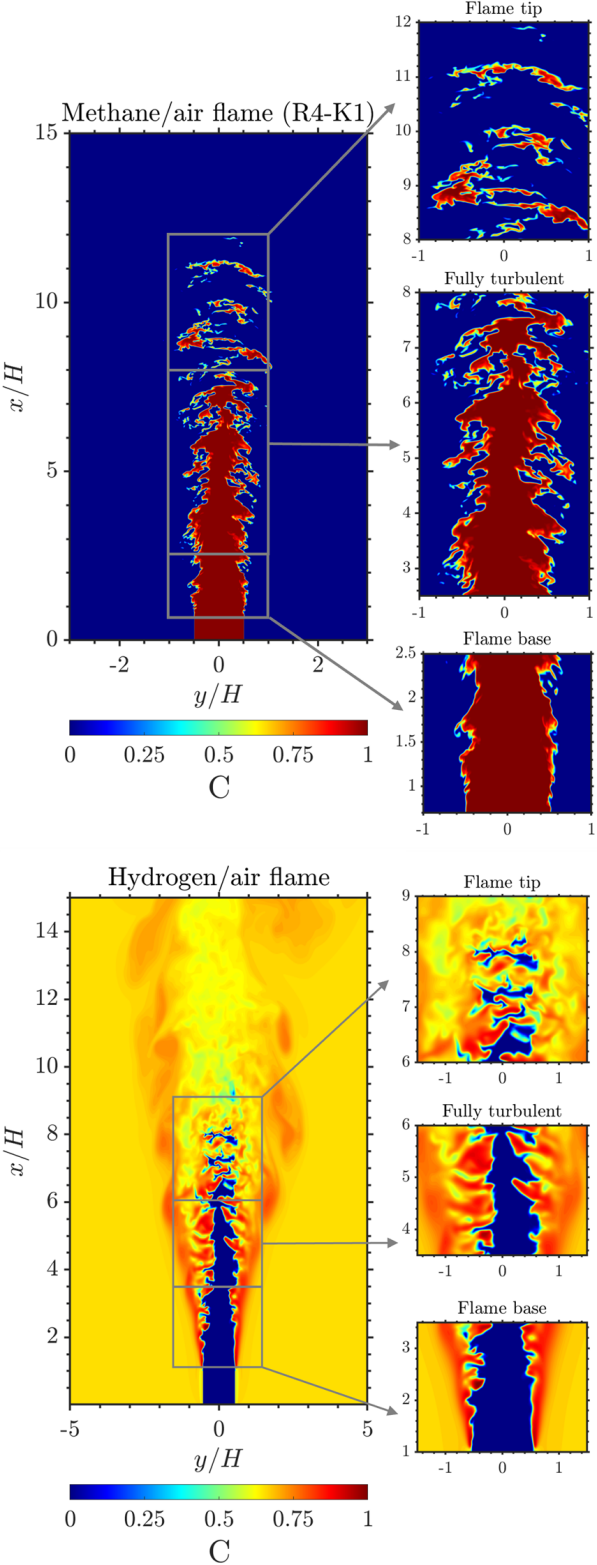
2D contour of the progress variable field showing the three different sub-regions: flame base, fully turbulent region, and the flame tip of the methane/air (R4-K1) flame (top row) and the hydrogen/air flame (bottom row)

Before the DNS is filtered, the fully developed turbulent sub-region of the DNS is first extracted. Figure 4 shows the three different regions, such as the flame base, the fully turbulent region, and the flame tip of the methane/air (R4-K1) and the hydrogen/air flames. In addition, either side of the flame is a coflow of fully burned gas which contains no information about the flame structure, therefore, it will not be used to train the models. The data extracted from the three sub-regions of the methane/air flames are used to train four models using each of the four DNS flames (R1-K1 to R4-K1) and are tested in all four flames. These models were trained and tested with DNS data filtered with only a filter-size ratio of 8. This is to study the extrapolation performance of the models when applied to different Reynolds numbers without the effects of different filter sizes or filter kernels.

To study the effects of filter size and kernel, both methane/air (R4-K1) and hydrogen/air datasets are employed. The models are only trained with data from the fully turbulent region. The DNS is filtered with filter sizes in the range of $n*dx$ using both box and Gaussian filters, where *dx* is the grid size of the DNS and *n* is the filtering factor in the range $\{n \in \mathbb {Z}: 2 \le n \le 16\}$. From hereon, the filter size will simply be referred to by its filtering factor. Due to the wide range of filtered data used to train the CNN models, a specific naming convention is used when referring to a particular model. For example, a model named *Box(2,4,8,16)* refers to a model that was trained with box-filtered data with specific filter sizes, while a model that was trained with box-filtered data with all filter sizes within the range of *n* will be *Box(2,3,4,5,6,7,8,9,10,11,12,13,14,15,16) *$\rightarrow$ *Box(2 to 16) *for brevity. The following models were trained and tested:

i. Four models trained with box filtered data: ${Box(2\,to\,16)}$, *Box(2,4,6,8,10,12,14,16)*, *Box(2,4,8,16)* and *Box(2,4,8)* and tested against all the filter sizes in the range of 2 to 16.
ii. Four models trained with Gaussian filtered data: ${Gaussian(2\,to\,16)}$, *Gaussian(2,4,6,8,10,12,14,16)*, *Gaussian(2,4,8,16)* and *Gaussian(2,4,8)* and tested against all the filter sizes in the range of 2 to 16.
iii. Four models trained with box filtered data with an increasing amount of training data, to study the convergence of the results with the increase in training data.
iv. Three models trained with both filter kernels: ${Box/Gaussian(2\,to\,16)}$, *Box/Gaussian(2,4,6,8,10,12,14,16)* and *Box/Gaussian(2,4,8,16)*.
v. A box-filter model trained with a weighted training data distribution. The amount of training data is increased with the increase in filter size.

After the DNS data has been filtered with a given filter size and kernel, the data has to be normalised before training the CNN. Normalising the data is crucial in ML as it ensures that the features used to train the models are on a similar scale which prevents certain features from dominating the learning process due to their larger size. This improves the training time and performance of the CNN models. In this study, the data is scaled to range between 0 and 1. The input field $\overline{C}$ is already in this range and therefore, does not need to be normalised. However, the output field $\overline{\Sigma }$ or $\overline{\dot{\omega }}_{\mathrm {H_2O}}$ does not range between 0 and 1 and therefore, must be normalised. This is achieved by dividing the output field by the maximum value of the output field in the DNS dataset, which ensures that any value in the output field is within the range of 0–1.

To train the CNN models, the patch-to-patch training strategy is adopted (Nista et al. 2024), i.e., 3D blocks of size $16\times 16\times 16$ cells are first extracted from the filtered dataset. The data blocks are extracted randomly and augmented by applying random $90^\circ$ rotations and/or flipping the data around a random axis. The data augmentation creates new variations of the original samples, which increases the diversity of the dataset and has been shown to improve the generalisability and overfitting of the CNN models (Lapeyre et al. 2019). A batch size of 50 blocks is used, with 40 blocks (80%) for training and 10 blocks (20%) for validation. The ADAM (Kinga et al. 2015) optimiser is used with a Mean Squared Error (MSE) loss function based on single cell values to optimise the weights. A total of 100 training steps is employed followed by 50 validation steps. In this study, a maximum of 200 epochs is prescribed to ensure sufficient convergence of the training. The total training time for 200 epochs is approximately 20 min on a single NVIDIA Volta 100 GPU.

## In-Sample Model Validation

**Fig. 5:**
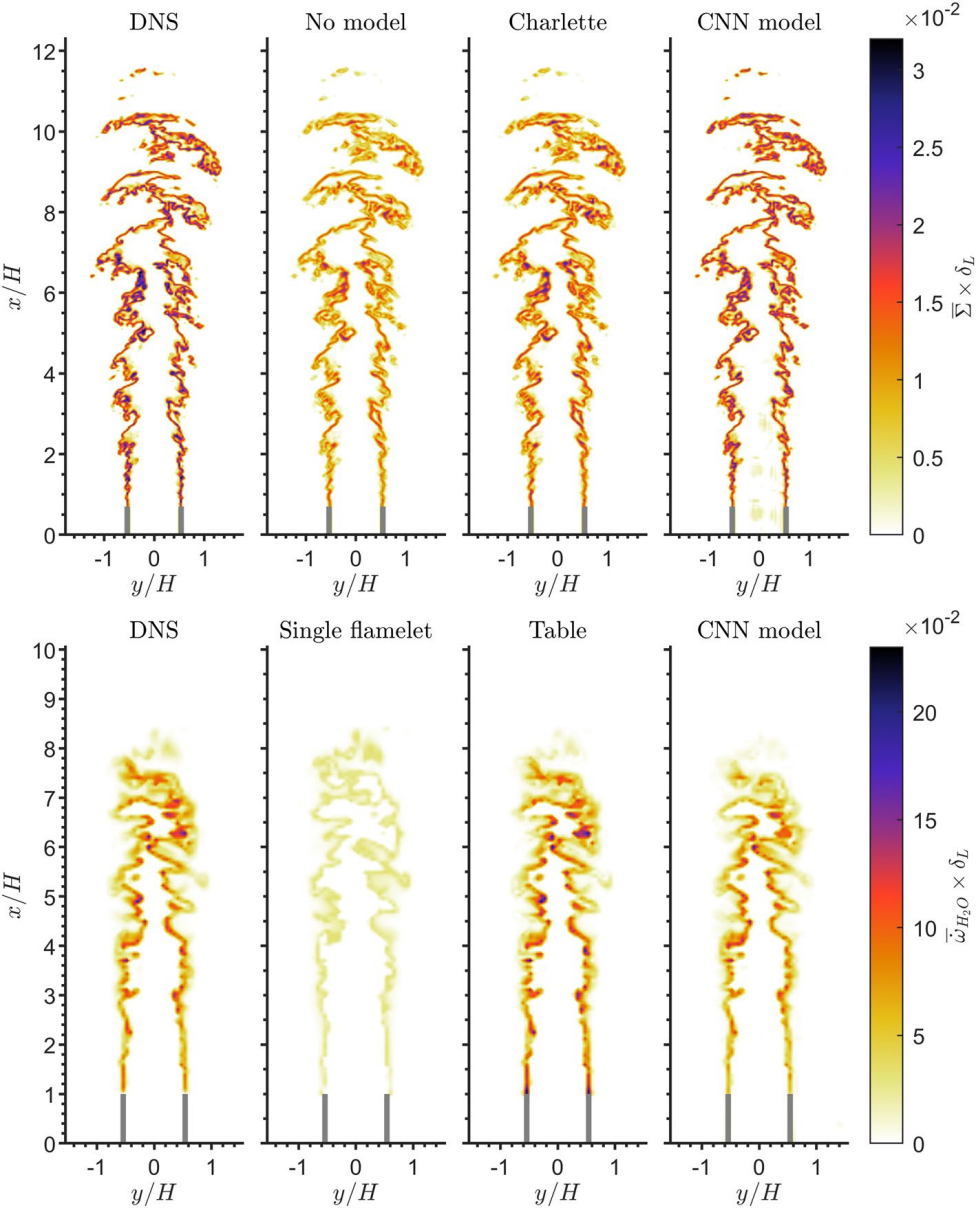
Top row: 2D contours of the filtered flame surface density $\bar{\Sigma }$ of the methane/air flame (R4-K1) from three different models compared against the DNS. Bottom row: 2D contours of the filtered progress variable source term $\overline{\dot{\omega }}_{\mathrm {H_2O}}$ of the hydrogen/air flame from three different models compared against the DNS. Both flames are filtered with a box filter of size 8

**Fig. 6:**
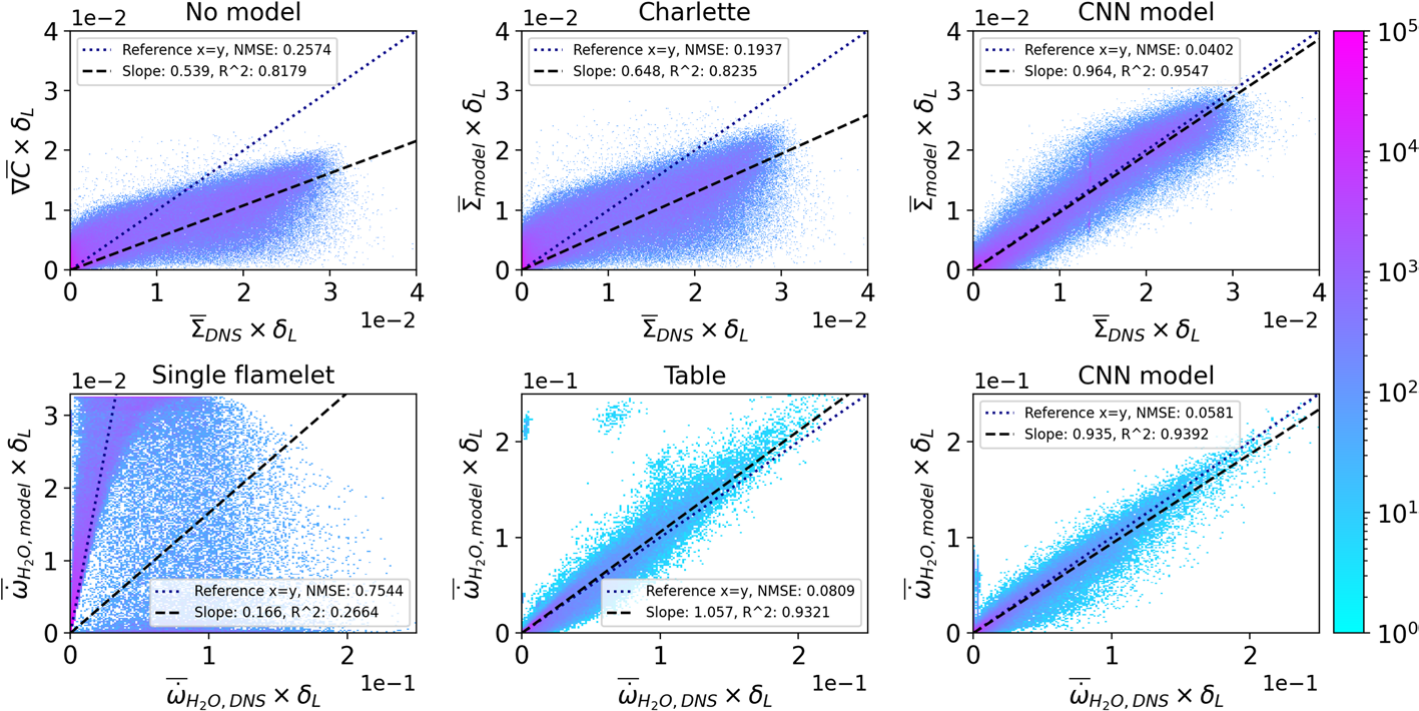
Top row: JPDFs of the filtered flame surface density $\bar{\Sigma }$ of the methane/air flame (R4-K1) from three different models compared against the DNS. Bottom row: JPDFs of the filtered progress variable source term $\overline{\dot{\omega }}_{\mathrm {H_2O}}$ of the hydrogen/air flame from three different models compared against the DNS. Both flames are filtered with a box filter of size 8

To validate the performance of the CNN model, in-sample testing is employed where both the training and testing data have the same flow conditions (e.g., same Re) and filter operator (i.e., same filter size and kernel). Figure 5 (top row) shows the 2D contours of $\overline{\Sigma }$ predicted by the CNN model compared against the DNS results filtered with a box filter of size 8. In addition, Fig. 5 (top row) also shows $\overline{\Sigma }$ obtained from the Charlette model, together with the flame surface density calculated without any SGS contributions, which in this context is simply the magnitude of the gradient of the filtered progress variable (i.e. $|\nabla \overline{C}|$) and is labelled as“No model”. A more detailed quantitative comparison between the model predictions and the DNS are also shown by the Joint Probability Density Functions (JPDF) in Fig. 6 (top row).

Without using an SGS model, the flame surface density is noticeably underpredicted. This underprediction worsens as the filter size is increased and the SGS contributions become more important. The validation results for a larger filter size of 16 can be found in the supplementary material. The Charlette model shows an improvement in the results where the model is able to capture some of the SGS contributions. The CNN model shows comparable performance to this specific implementation of the Charlette model and even performs slightly better in certain locations. The CNN model is able to reproduce the $\overline{\Sigma }$ field, which agrees well with the DNS results in terms of both the magnitude and the spatial distribution. This is confirmed by the JPDF which shows predictions align closely with the DNS results with a relatively low scatter along the regression line, as shown by the low Normalised Mean Squared Error (NMSE) and a high coefficient of determination ($R^2$) values.

Figure 5 (bottom row) shows the 2D contours of $\overline{\dot{\omega }}_{\mathrm {H_2O}}$ obtained from the CNN model and two additional flamelet-based models compared with $\overline{\dot{\omega }}_{\mathrm {H_2O}}$ obtained from the hydrogen/air DNS filtered with a box filter of size 8. A more detailed quantitative comparison between the model predictions and the DNS are also shown by the JPDF’s in Fig. 6 (bottom row).“Single flamelet” refers to $\overline{\dot{\omega }}_{\mathrm {H_2O}}$ obtained from a single 1D unstretched laminar flamelet. Whereas,“Table”refers to $\overline{\dot{\omega }}_{\mathrm {H_2O}}$ obtained from a flamelet table constructed using a collection of 1D unstretched laminar flamelets as described in Sect. 3.2. The single flamelet model shows poor performance where $\overline{\dot{\omega }}_{\mathrm {H_2O}}$ is significantly underpredicted. The corresponding JPDF shows the model significantly underpredicts $\overline{\dot{\omega }}_{\mathrm {H_2O}}$ which leads to a relatively high NMSE. There is also a large scatter in the predictions, resulting in a low $R^2$ value. The single flamelet model, which is often used for modelling turbulent premixed flames, is unable to capture the enhanced reaction rates encountered in the thermodiffusivly unstable hydrogen/air flame (Berger et al. 2022; Regele et al. 2013). Furthermore, the single flamelet predicts $\overline{\dot{\omega }}_{\mathrm {H_2O}}$ to be constant along the flame front and therefore, does not capture the local variations of $\overline{\dot{\omega }}_{\mathrm {H_2O}}$ along the flame front. In comparison, the flamelet table, which takes into account the variations of the mixture fraction in the flame, is able to significantly improve the accuracy of the predictions. The $\overline{\dot{\omega }}_{\mathrm {H_2O}}$ obtained from the flamelet table agrees well with the DNS results and shows a significant improvement over the single flamelet model. The corresponding JPDF shows that the predictions are in close agreement with the DNS results, with minimal scatter along the regression line. A similar level of performance is observed with the CNN model. The JPDF of the CNN model is very similar to that of the flamelet table. The predictions of the CNN model align very closely with the DNS results with minimal scatter along the regression line. However, the CNN model provides slightly more accurate predictions with a lower NMSE than the flamelet model. With only the progress variable field as the input to the CNN model, the model is able to accurately map the intricate structures of the $\overline{\dot{\omega }}_{\mathrm {H_2O}}$ field, which closely matches the DNS. Overall, this qualitative comparison shows that the CNN model is adequately trained and performs extremely well under in-sample test conditions.

## Effect of Reynolds Number

To assess the performance of the CNN models to generalise at different Reynolds numbers, each of the four methane/air flames shown in Table 1 has been used to train a CNN model. Then each of these models was applied to each of the flames to predict the flame surface density. To eliminate any effects of filter size and kernel, data filtered with only a box filter size ratio of 8 was used to train these models. More importantly, since the Kolmogorov scale is approximately constant in all the flames, the ratio $\Delta /\eta$ is conserved in all training and testing data. A previous study by Nista et al. (2023) reported that conserving the $\Delta /\eta$ ratio is fundamental to achieving generalisation capabilities for different flow configurations.

**Fig. 7:**
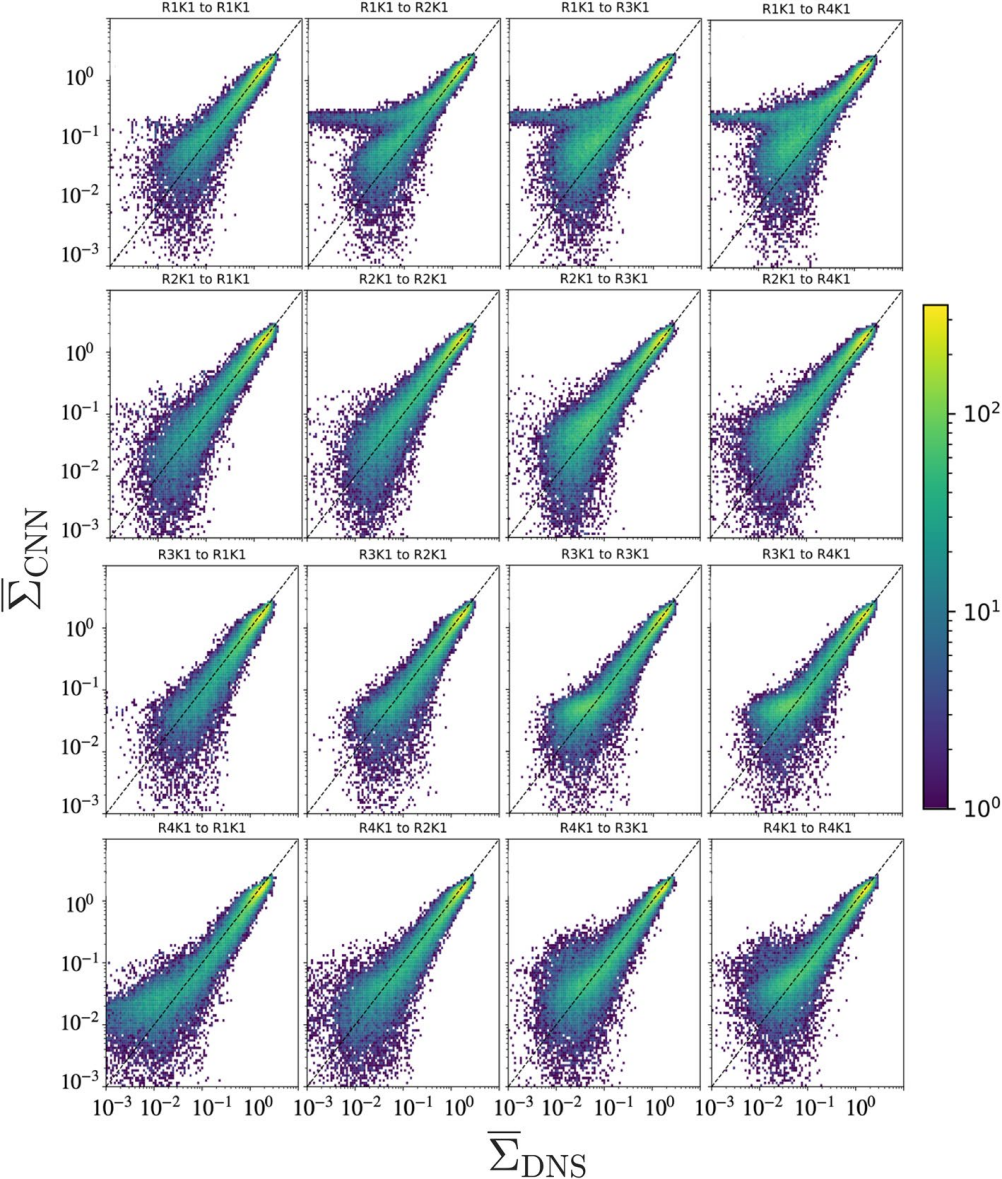
JPDFs of the flame surface density $\overline{\Sigma }$ for CNN models trained and applied to flames at different Reynolds numbers. From top to bottom, the Reynolds number of the training data increases; from left to right, the Reynolds number of the data used for testing increases

The JPDFs of $\overline{\Sigma }$ from the DNS and the values predicted by the CNN are shown in Fig. 7. The performance is generally good, with an overwhelming probability of errors below 20% for large values of $\overline{\Sigma }$ in the DNS. For very small values, the probability of discrepancies is slightly higher. While the overall behaviour shown in this assessment does not show large qualitative differences for the CNN trained at a certain Reynolds number and applied to a different one, it can be observed that the model trained with low Reynolds number data (R1-K1), when applied to the high Reynolds number case (R4-K1) is characterised by an overall overprediction with respect to $\overline{\Sigma }$ in the DNS, as shown in the top right panel. A similar, albeit less evident, behaviour is observed for the same model when applied to R3-K1 and for the model trained with R2-K1 data when applied to the high-Re cases R3-K1 and R4-K1. On the other hand, the model trained on R3-K1 performs better when applied to R4-K1.

**Fig. 8:**
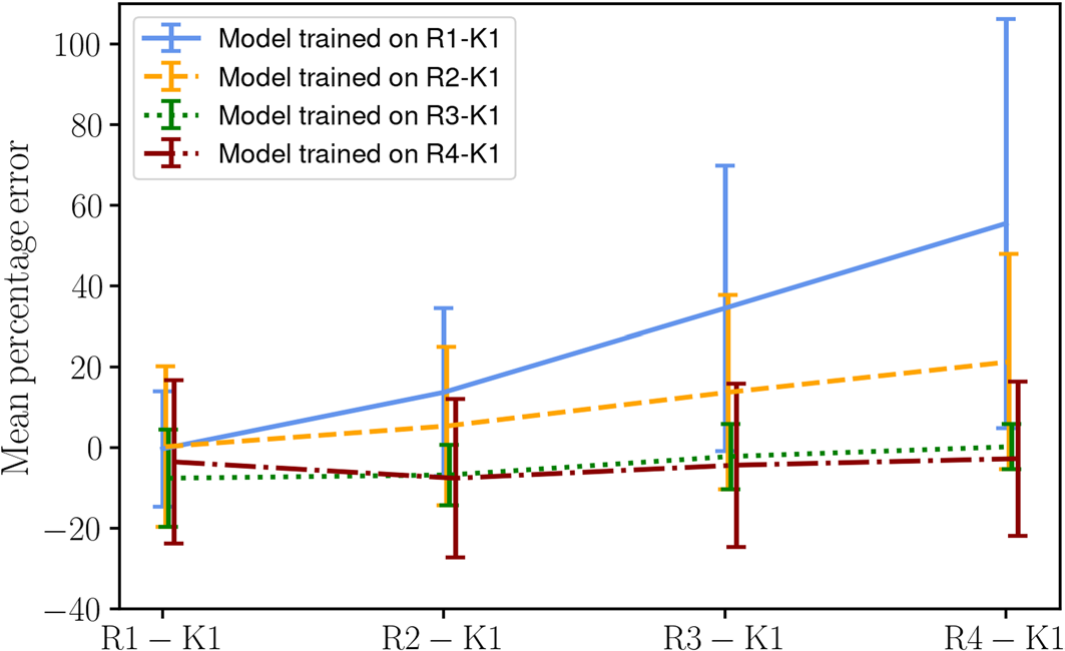
Mean percentage error for CNN models trained and applied to flames at different Reynolds numbers. The error bars show the $25^{th}$ and $75^{th}$ percentiles of the percentage error

To further quantify the extrapolation performance of the models, Fig. 8 shows the mean and the $25^{th}$ and $75^{th}$ percentiles of the percentage error between the DNS and the CNN prediction for all the cases shown in Fig. 7. Each of the lines in the figure shows how a model trained on a particular flame performs when tested with data from other flames. While the models trained with data from the two lowest Reynolds number cases (R1-K1 and R2-K1) perform well when applied to the low Reynolds number cases and progressively worse when applied to higher Reynolds number flames, the model trained with high Reynolds number data appears to have achieved asymptotic behaviour and the overall error is independent of the case to which the model is applied. In particular, it is evident that the models trained on R3-K1 and R4-K1 have the same error when applied to data from R4-K1, while the models trained on R1-K1 and R2-K1 are further away from the DNS values when applied to R4-K1. This suggests that generalisation to higher Reynolds numbers is possible provided the training data is collected at a Reynolds number high enough to approach the asymptotic state of high-Reynolds number turbulence. This observation is also consistent with previous analyses of the same flame series (Luca et al. 2019; Attili et al. 2021) that showed that the low-Re cases tend to deviate from the asymptotic behaviour of the high-Re flames.

## Effect of Filter Size

A typical LES mesh contains cells with a range of filter sizes and the exact filter size is usually unknown *a priori*, due to the combined effects of numerical errors and implicit grid filtering. Therefore, it is important that a CNN model that has been trained with a certain set of filter sizes is able to be applied across a wide range of filter sizes that have not been used to train the model. In this section, several CNN models are trained with data filtered with a range of specific filter sizes, and the models are tested on data filtered with all filter sizes from 2 to 16. It is worth noting that the filter kernel used to filter the training data is the same as that used for the testing data, e.g., a box-filtered model is always tested with box-filtered test data. This is done to avoid any effects of the filter kernel, which are studied separately in Sect. 8. All models are trained with 100 blocks of data for each filter size used.

**Fig. 9:**
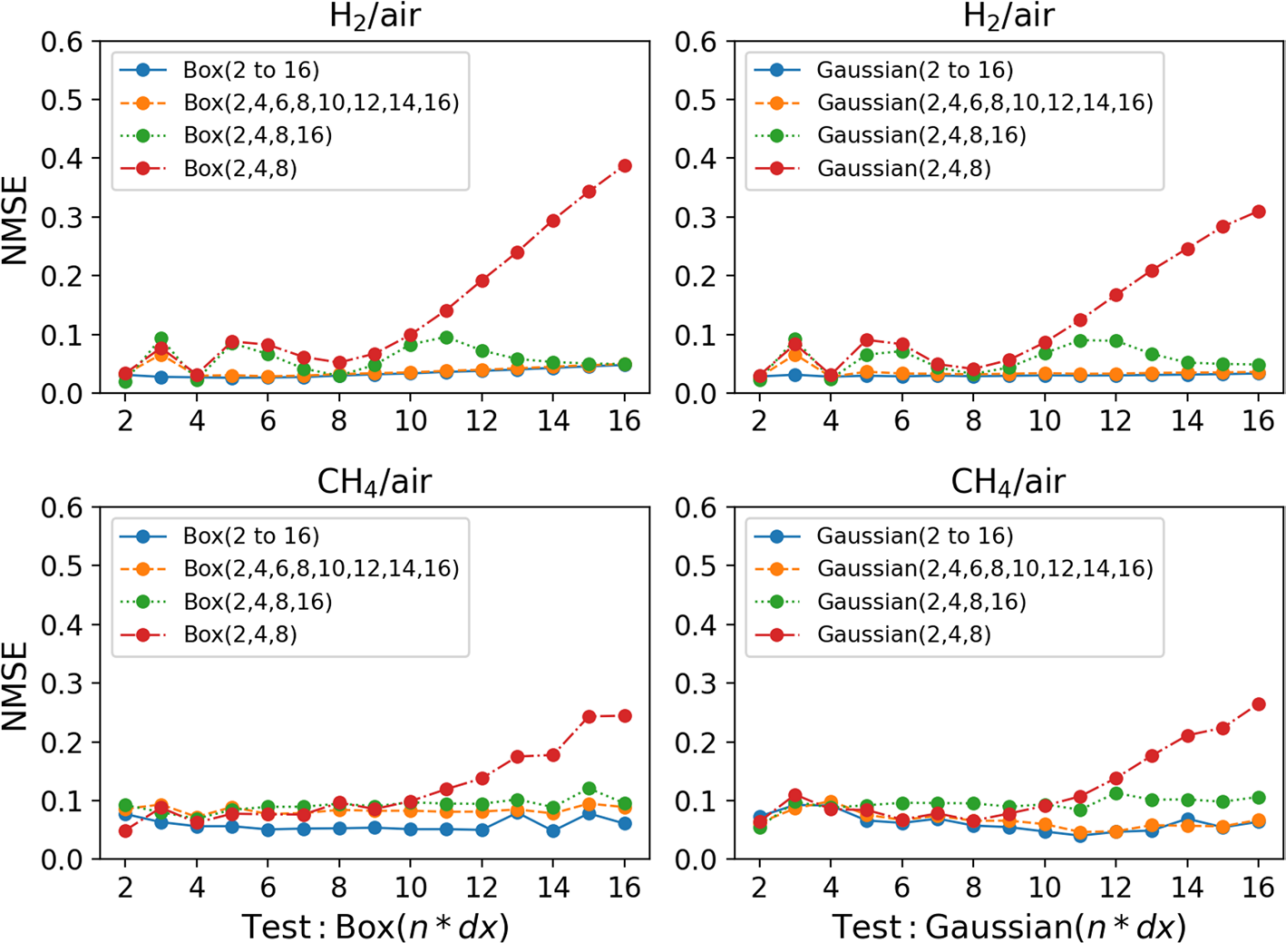
Top row: NMSE for CNN models applied to a range of filter sizes predicting the progress variable source term $\overline{\dot{\omega }}_{\mathrm {H_2O}}$ trained using the hydrogen/air flame. Bottom row: NMSE for CNN models applied to a range of filter sizes predicting the flame surface density $\overline{\Sigma }$ trained using the methane/air flame (R4-K1)

Figure 9 shows the performance of different CNN models trained with either box or Gaussian filter kernels with different filter sizes. The models show excellent interpolation performance when applied to filter sizes that were not used to train the model. The models are able to interpolate between filter sizes with good accuracy for both box and Gaussian filtered models. Even models trained with few filter sizes, such as the *Box(2,4,8,16)* model, perform very similarly to the model containing all filter sizes ${Box(2\,to\,16)}$. This shows that training data with only a few filter sizes is sufficient to train a CNN model with good performance. This significantly reduces the amount of training data required to train the models. In addition, the interpolation performance of the CNN models is very similar for the hydrogen/air flame and the methane/air flame. This further indicates that the CNN models are capable of learning complex features embedded in the unstable hydrogen/air flame with similar accuracy to predictions in the methane/air flame.

**Fig. 10:**
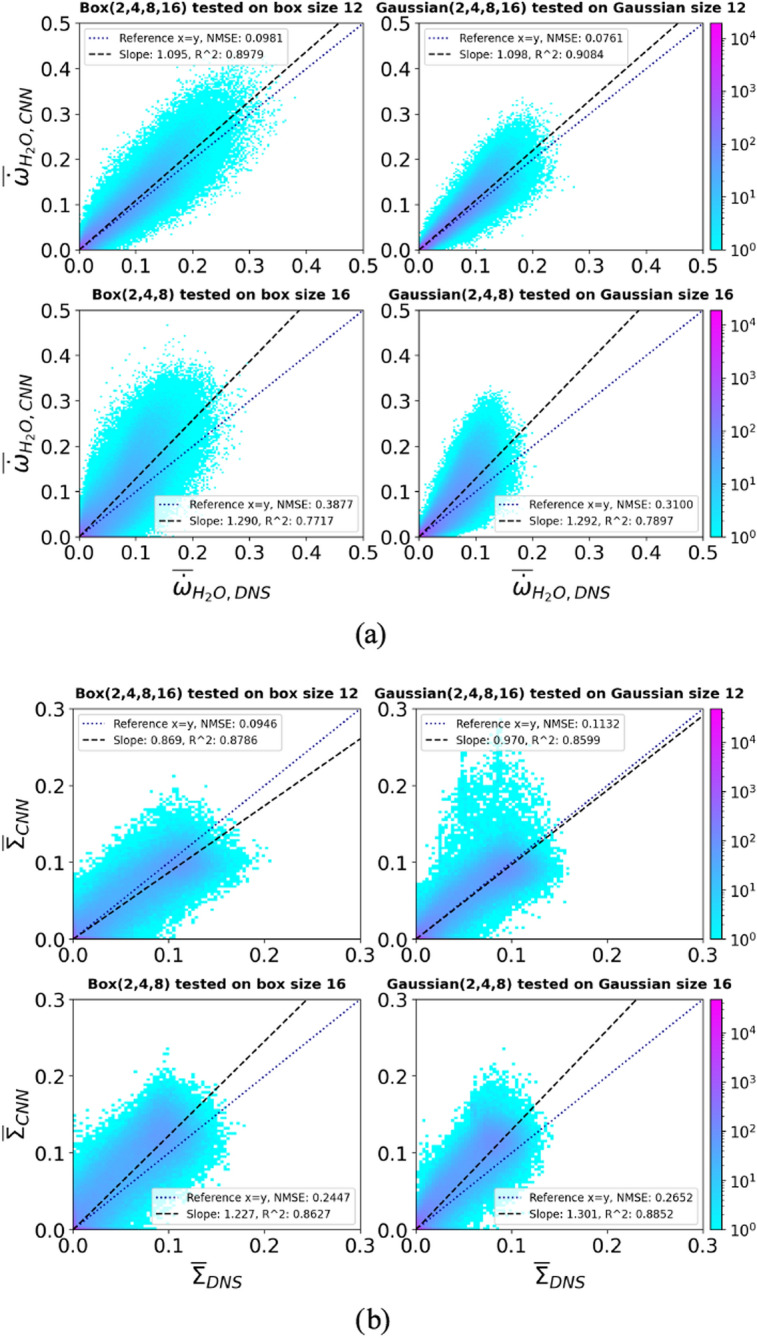
JPDFs between the DNS and the predicted values for CNN models trained with a specific number of filter sizes and tested on filter sizes that were not used for training.** a** JPDFs of the progress variable source term $\overline{\dot{\omega }}_{\mathrm {H_2O}}$ between the DNS and CNN predictions. Training data is taken from the hydrogen/air flame.** b** JPDFs of the flame surface density $\overline{\Sigma }$ between the DNS and CNN predictions. Training data is taken from the methane/air flame (R4-K1).

Even though the models show excellent interpolation performance, the extrapolation performance is slightly less accurate. When the *Box(2,4,8)* or *Gaussian(2,4,8)* model is tested with filter sizes larger than 8, the performance gets progressively worse as the filter size increases, as shown in Fig. 9. This increase in error is slightly worse for the hydrogen/air flame. Unlike the interpolation, when it comes to the extrapolation performance, it appears that the complexity of the predicted features makes extrapolation more difficult at larger filter sizes. Although the NMSE gets progressively worse as the filter size increases, the overall extrapolation performance is at an acceptable level. As shown in Fig. 10, the JPDFs of the *Box(2,4,8)* and *Gaussian(2,4,8)* models tested with a filter size of 16 show that the predicted results still agree relatively well with the DNS results for both $\overline{\dot{\omega }}_{\mathrm {H_2O}}$ and $\overline{\Sigma }$ predictions. For comparison, the *Box(2,4,8,16)* and *Gaussian(2,4,8,16)* models tested with filter size 12, where the predictions have to be interpolated, are also shown in Fig. 10. The JPDFs of these two models are similar to the JPDFs of the *Box(2,4,8)* and *Gaussian(2,4,8)* models tested with a filter size of 16. A slightly larger scatter of the data and slight deviations from the equality line lead to a higher NMSE for the *Box(2,4,8)* and *Gaussian(2,4,8)* models. Overall, the extrapolation performance is comparable to the interpolation performance. Therefore, these *a priori* tests indicate that a CNN model trained with a selected number of filter sizes can be applied across a wide range of filter sizes with good overall performance.

## Effect of Filter Kernel

Apart from having a range of filter sizes, a typical LES mesh contains cells with unknown shapes that implicitly filter the solution differently. Each of these LES cells acts as a filter kernel with a certain size and information about the kernel type is usually unknown. Therefore, it is important that a CNN model trained with data filtered with a certain filter kernel can also be applied to data filtered with a different filter kernel. In this section, data from the $\mathrm H_2/air$ flame is used to predict the progress variable source term and the $\mathrm CH_4/air$ flame with the R4-K1 configuration to predict the flame surface density. The same datasets are used to both train and test the CNN models. For example, a model trained with R4-K1 data is tested with R4-K1 data. Therefore, the training and testing data are statistically similar, but the testing data was never seen by the CNN model during training. The CNN models are trained using data filtered with a range of filter sizes and a certain filter kernel. The models are then tested on data filtered with a different filter kernel. For example, a model trained with only box-filtered data is tested on Gaussian-filtered data and vice versa to study the extrapolation performance of the model to different filter kernels.

**Fig. 11:**
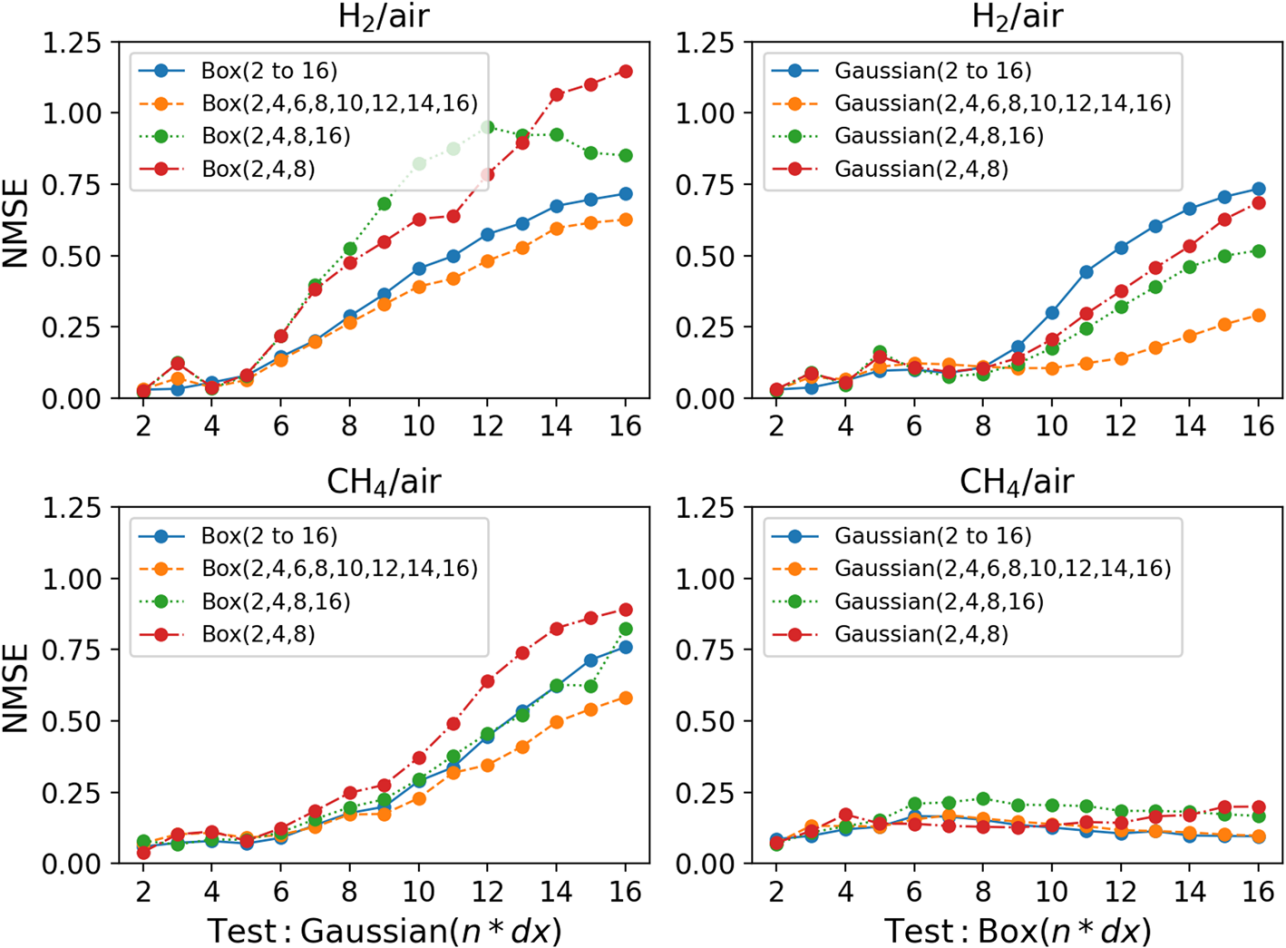
Top row: NMSE for CNN models applied to data filtered with a different filter kernel than the one used to train the model. The models predict the progress variable source term $\overline{\dot{\omega }}_{\mathrm {H_2O}}$ trained with data from the hydrogen/air flame. Bottom row: NMSE for CNN models tested on data filtered with a different filter kernel than the one used to train the model. The models predict the flame surface density $\overline{\Sigma }$ trained with data from the methane/air flame (R4-K1)

Figure 11 (top row) shows the performance of the CNN models trained with data filtered with a certain filter kernel and tested with data filtered with a different filter kernel to predict the progress variable source term in the hydrogen/air flame. When the CNN models trained with box-filtered data only (hereafter referred to as a box model) are applied to Gaussian-filtered data and vice versa, the accuracy of the predictions is very good at small filter sizes. However, the predictions of all tested models get progressively worse as the filter size is increased. The accuracy of the predictions is consistent at smaller filter sizes up to a filter size of about 5 when the box models are tested with Gaussian-filtered data. Thereafter an increase in inaccuracy can be observed. However, this increase is delayed when models that were trained with Gaussian-filtered data (hereafter referred to as Gaussian models) are tested with box-filtered data (Fig. 11, top right). The deterioration of the predictions starts around a filter size of 9 and the overall increase in the NMSE is lower than for the box models tested against Gaussian data. This difference is even more evident in the results for the flame surface density predictions in the R4-K1 methane/air flame, which are shown in Fig. 11 (bottom row). In the methane/air flame, there is a significant improvement in the results when the Gaussian models are tested with box-filtered data. Unlike the $\overline{\dot{\omega }}_{\mathrm {H_2O}}$ predictions, the NMSE does not increase with the increase in filter size. The accuracy of the predictions is consistent for all tested filter sizes. However, the $\overline{\Sigma }$ predictions of the box models tested with Gaussian-filtered data show similar performance to the $\overline{\dot{\omega }}_{\mathrm {H_2O}}$ predictions from the same type of models. This shows that the CNN models trained with box-filtered data perform very similarly in both flames (R4-K1 and hydrogen/air flame). Conversely, the Gaussian models perform better across both flames. More importantly, a significant improvement is observed when the models are applied to the methane/air flame (R4-K1).

For a given filter size, the Gaussian kernel filters more compared to a box filter kernel. The JPDFs that show this difference in filtering can be found in the supplementary material. However, when the filter size is small, the box and Gaussian filters behave very similarly. The amount of information filtered by both models becomes very similar. Therefore, the CNN models trained with either box or Gaussian-filtered data show similar performance at small filter sizes. As the filter size increases, the difference between the two filter kernels increases and a difference in the NMSE of the predictions is observed.

**Fig. 12:**
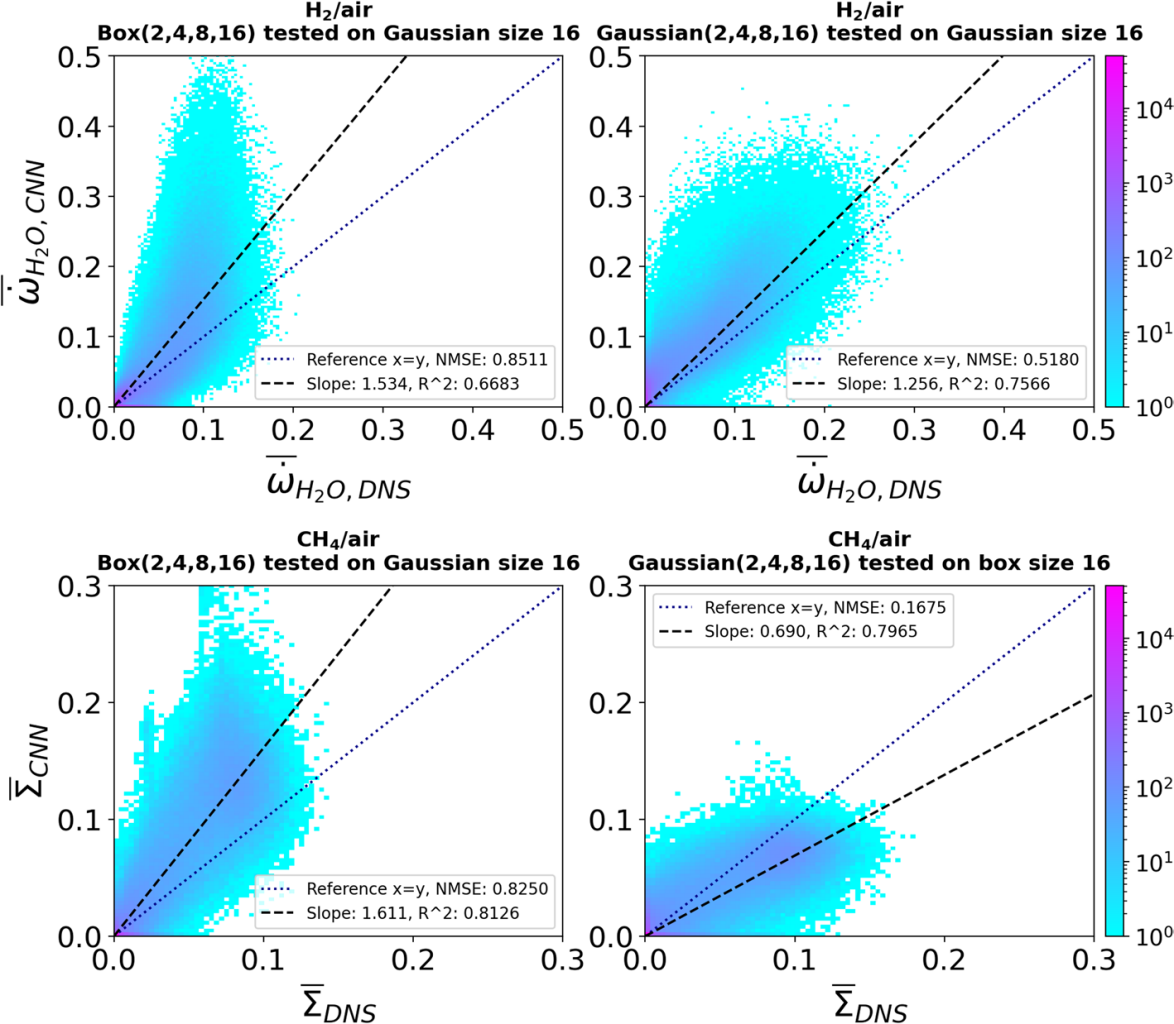
Top row: JPDFs between the DNS and predicted values for CNN models trained on data filtered with a certain filter kernel and applied to data filtered with a different filter kernel. The models predict the progress variable source term $\overline{\dot{\omega }}_{\mathrm {H_2O}}$ trained using the hydrogen/air flame. Bottom row: JPDFs between the DNS and the predicted values for CNN models trained with data filtered from a certain filter kernel and applied to data filtered with a different filter kernel. The models are predicting the flame surface density $\overline{\Sigma }$ trained with the methane/air flame (R4-K1)

Figure 12 shows the JPDFs of the *Box(2,4,8,16)* model tested with data filtered with a Gaussian filter of size 16 and a box filter of size 16 for both methane/air (R4-K1) and hydrogen/air flames. The performance of the box model is very similar for both flames, with a very similar deviation of the regression line from the equality line (CNN output = DNS output). In addition, the box model mostly overpredicts the DNS value in both flames indicated by the slope of the regression line, which is above one. Whereas, the Gaussian model behaves slightly differently between the two flames. The Gaussian model applied to the methane/air flame underpredicts the DNS while it overpredicts the DNS in the hydrogen/air flame. Moreover, the regression line for the Gaussian model is slightly closer to the equality line in the hydrogen/air flame than in the methane/air flame. However, the NMSE in the hydrogen/air flame is slightly larger than in the methane/air flame. The same trend is also observed for the box models, as indicated by the lower $R^2$ value of the data scatter along the regression line in the hydrogen/air flame, resulting in higher NMSE values. Overall, the Gaussian models have better extrapolation performance into different filter kernels compared to the box models and are therefore, more suitable for ML applications.

**Fig. 13:**
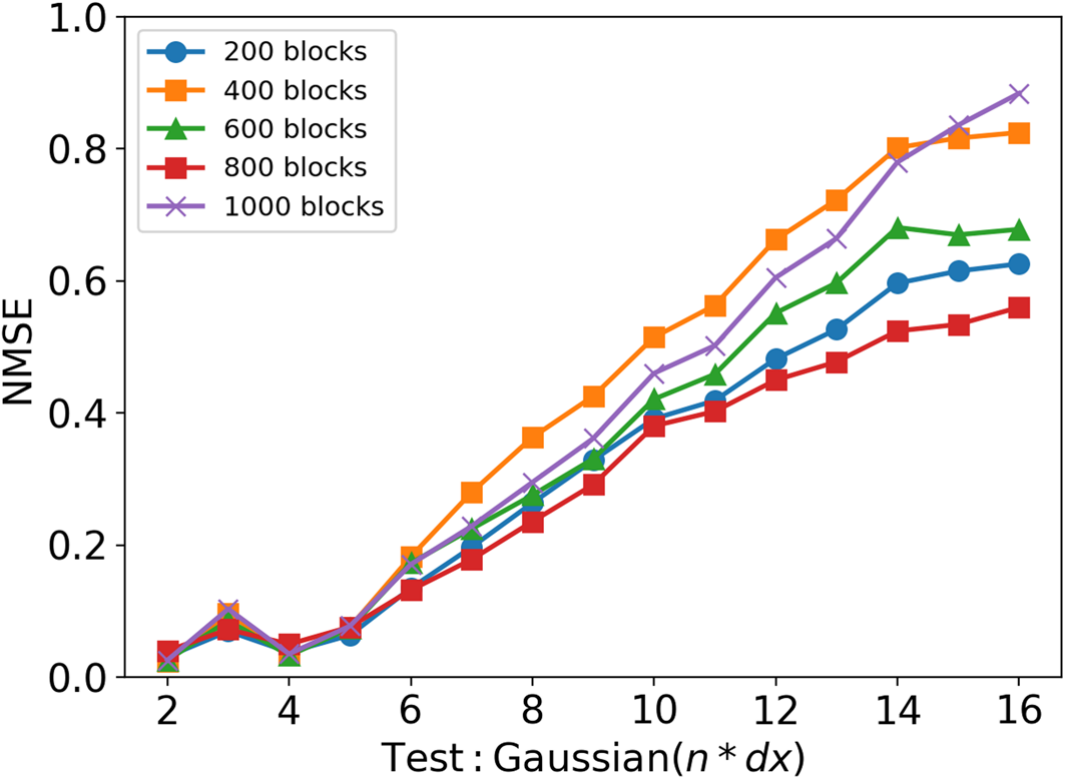
NMSE for the *Box(2,4,6,8,10,12,14,16)* model trained with increasing amount of training data. The model is predicting $\overline{\dot{\omega }}_{\mathrm {H_2O}}$

As the filter size increases, the amount of information being filtered increases. Therefore, each filtered block of training data contains less information about the flame structure or, conversely, there is more to learn for the field filtered with large filter sizes. Since the amount of training data per filter size is the same during training, the amount of training information for each filter size gets progressively smaller as the filter size increases, while the information to learn gets larger. An attempt to reduce this issue is to increase the amount of training data for the models. The aim is to add enough information for larger filter sizes and reduce the errors in the predictions. The amount of training data is gradually increased to study the convergence of the NMSE of the model predictions. Figure 13 shows the performance of a series of *Box(2,4,6,8,10,12,14,16)* models trained with an increasing amount of training data. The models predict $\overline{\dot{\omega }}_{\mathrm {H_2O}}$ in the hydrogen/air flame. Five models are trained starting with a volume of 200 data blocks per filter size and the amount of training data is increased in 200 blocks up to a maximum of 1000 blocks. It is worth noting that the number of data blocks is evenly distributed among the filter sizes so that each filter size has the same number of data blocks. The results show that the NMSE does not change for small filter sizes as the training data increases. This indicates that the training is converged for small filter sizes. However, as the filter size increases, a difference in the NMSE values is observed with the increase in training data, which does not show a monotonic trend. The NMSE values for larger filter sizes tend to fluctuate as the amount of training data increases. Therefore, they do not seem to be converging on a specific value. However, the qualitative trends are unchanged as the prediction errors get progressively worse as the filter size increases, regardless of the amount of training data.

**Fig. 14:**
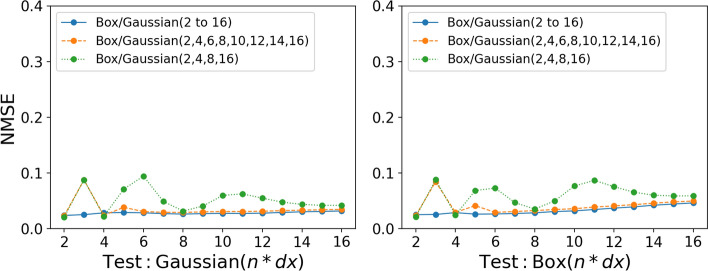
NMSE for CNN models trained with both box and Gaussian filtered data predicting $\overline{\dot{\omega }}_{\mathrm {H_2O}}$

Instead of training the CNN models with data filtered with a single type of filter kernel, both box and Gaussian filtered data can be used to train the models. Figure 14 shows the performance of the CNN models when trained with both box and Gaussian filtered data. All models show excellent performance when tested with both box and Gaussian filtered data across all the tested filter sizes. Moreover, a study by Nista et al. (2024) investigated the influence of training filter kernels on the reconstruction capability of super-resolution generative adversarial network (SR-GAN), based on CNNs, for turbulence closure. Three different filter kernels were used in their study: box, Gaussian and spectral filters. It was found that a model trained with both box and spectral filtered data performed slightly better when applied to Gaussian filtered data compared to a model trained only with either box or spectral filtered data. Therefore, mixing filter kernels during the training could improve the robustness of the CNN models and their ability to extrapolate to different filter kernels.

**Fig. 15:**
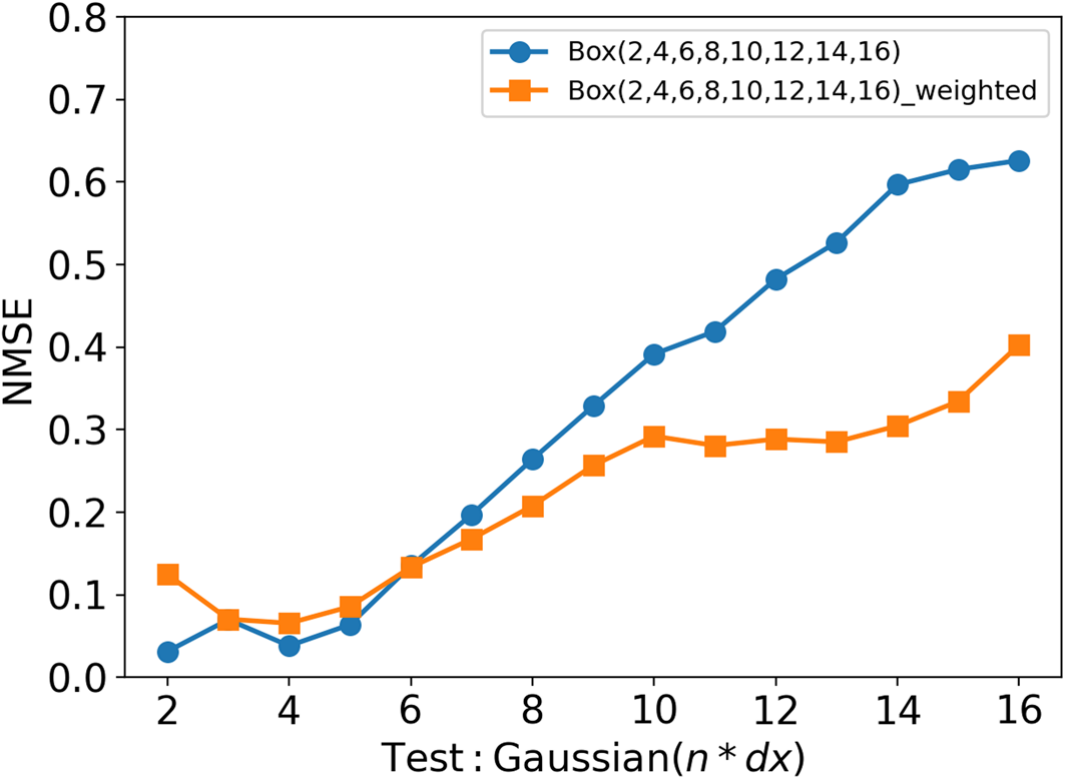
NMSE for a CNN model trained with a weighted data distribution across the filter sizes. A model with evenly distributed blocks of data (200 blocks of data per each filter size) is also shown for comparison. Both models are predicting $\overline{\dot{\omega }}_{\mathrm {H_2O}}$

Since the larger filter sizes contain less information about the flame structure, there is an imbalance of information across the filter sizes. Therefore, an additional CNN model is trained with a weighted data distribution. The larger filter sizes are weighted more heavily and vice versa for smaller filter sizes. The amount of training data for each filter size is gradually increased as the filter size increases. The aim is to approximately balance the level of information contained within each filter size. The amount of training data is doubled for each filter size used, starting with only 8 blocks of data for filter size 2 and ending with 1024 blocks for filter size 16. Figure 15 shows the performance of the weighted model compared to a model with uniformly distributed blocks of data, which is simply referred to as the unweighted model. The weighted model shows an improvement in predictions for larger filter sizes while maintaining good accuracy for smaller filter sizes. Even with only 8 blocks of training data for filter size 2, the accuracy of the predictions did not change significantly compared to the unweighted model. This indicates that the smaller filter sizes contain enough information so that the CNN model can be trained with relatively small amounts of training data to provide accurate predictions. Even though the weighted model performs better than the unweighted model at larger filter sizes, the NMSE of the predictions is still higher than that at low filter sizes. Even with 1024 blocks of filter size 16 data, the NMSE is still not comparable to the values found at lower filter sizes. However, the weighted model is a more robust CNN model that is much less dependent on the type of filter kernel compared to the unweighted model and therefore more suitable for LES.

## Conclusions

A CNN based on U-Net architecture is used to develop data-driven models for subgrid-scale flame wrinkling in methane/air premixed flames and the filtered progress variable source term in hydrogen/air premixed flames. These models are trained using filtered DNS data and tested to evaluate their extrapolation performance under various conditions. The performance was studied in terms of Reynolds numbers, filter sizes, and filter types that were not included in the training. The key conclusions are:

1. Models trained with data from the two lowest Reynolds number cases (R1-K1 and R2-K1) perform well when applied to the low Reynolds number cases and get progressively worse when applied to larger Reynolds number flames. The model trained with high Reynolds number data appears to have achieved asymptotic behaviour and the overall error is independent of the case to which the model is applied. This suggests that generalisation to higher Reynolds numbers is possible, provided that the training data is collected at a Reynolds number high enough to approach the asymptotic state of high-Reynolds number turbulence.
2. When the CNN models are tested with the same type of filter used for training, they show excellent performance. Furthermore, the model is able to interpolate accurately between different filter sizes. Even models trained with few filter sizes are able to interpolate with relatively small errors. The extrapolation performance of the models is not as good as the interpolation, but is at an acceptable level. This indicates that models trained with only a few filter sizes can be successfully applied across a wide range of filter sizes.
3. When the CNN models trained with box filter type data are applied to Gaussian-filtered data and vice versa, the model performs well with smaller filter sizes. However, the performance gets progressively worse as the filter size increases. Increasing the amount of data does not improve the results. However, mixing box and Gaussian filtered data during training significantly improved the results. In addition, using a weighted distribution of the training data (i.e. gradually increasing the amount of training data with increasing filter size) without mixing the filter types during training shows an improvement in results compared to a model with uniformly distributed training data. This leads to a more robust CNN model that is less dependent on the filter type and better suited for an LES.

## Supplementary Information

Below is the link to the electronic supplementary material.

Supplementary file1 (PDF 4966 KB)
