# Supplementary material for: Extrapolation Performance of Convolutional Neural Network-Based Combustion Models for Large-Eddy Simulation: Influence of Reynolds Number, Filter Kernel and Filter Size
Source: Flow Turbul Combust. 2025 Mar 24;115(3):1261–90. doi: 10.1007/s10494-025-00643-w (PMC12507949; doi:10.1007/s10494-025-00643-w)
Supplement: Supplementary file 1 — Supplementary file1 (PDF 4966 KB) [file 10494_2025_643_MOESM1_ESM.pdf]

Extrapolation performance of convolutional neural network-based combustion models for large-eddy simulation: Influence of Reynolds number, filter kernel and filter size
Supplementary material

Geveen Arumapperuma[1*], Nicola Sorace[1], Matthew Jansen[1], Oliver Bladek[1], Ludovico Nista[2], Shreyans Sakhare[2], Lukas Berger[2], Heinz Pitsch[2], Temistocle Grenga[3], Antonio Attili[1]

[1*]School of Engineering, The University of Edinburgh, Edinburgh, EH8 3JL, Scotland, UK.
[2]Institute for Combustion Technology, RWTH Aachen University, Aachen, 52056, Germany.
[3]Faculty of Engineering and Physical Sciences, University of Southampton, Southampton, SO17 1BJ, United Kingdom.

*Corresponding author(s). E-mail(s): geveen.arumapperuma@ed.ac.uk;
Contributing authors: s1645762@sms.ed.ac.uk; m.g.d.jansen@sms.ed.ac.uk; o.bladek@sms.ed.ac.uk; l.nista@itv.rwth-aachen.de; s.sakhare@itv.rwth-aachen.de; l.berger@itv.rwth-aachen.de; h.pitsch@itv.rwth-aachen.de; t.grenga@soton.ac.uk; antonio.attili@ed.ac.uk;

1 Charlette model with different β values

Figure S1 shows the Joint Probability Density Function (JPDF) of the filtered flame surface density of the methane/air (R4-K1) flame calculated using the Charlette model with different β values. With a filter size of 8, all the models show relatively good overall performance. The NMSE does not change significantly with the change in the β value. Nevertheless, with a filter size of 8, the model with $\beta = 1.2$ is the best

performing model with the lowest NMSE value. However, with a filter size of 16, the performance of the models differs significantly with the change in the β value. At low β values, the models significantly underpredict the flame surface density while at high β values, the models significantly overpredict the flame surface density. The model with $\beta = 1.2$, which has the best performance with a filter size of 8, is no longer the best performing model with a filter size of 16. The best performing model at filter size 16 is the model with $\beta = 0.5$, which has the lowest NMSE of all models tested. For both filter sizes, the model with $\beta = 0.5$ shows similar performance, while the model with $\beta = 1.2$ shows significant differences. The NMSE of the model with $\beta = 1.2$ differs significantly between the two filter sizes. The NMSE has increased by a factor of 3.6 for filter size 16 compared to filter size 8. The model with $\beta = 0.5$ shows good overall performance across both filter sizes and is, therefore, a more robust model.

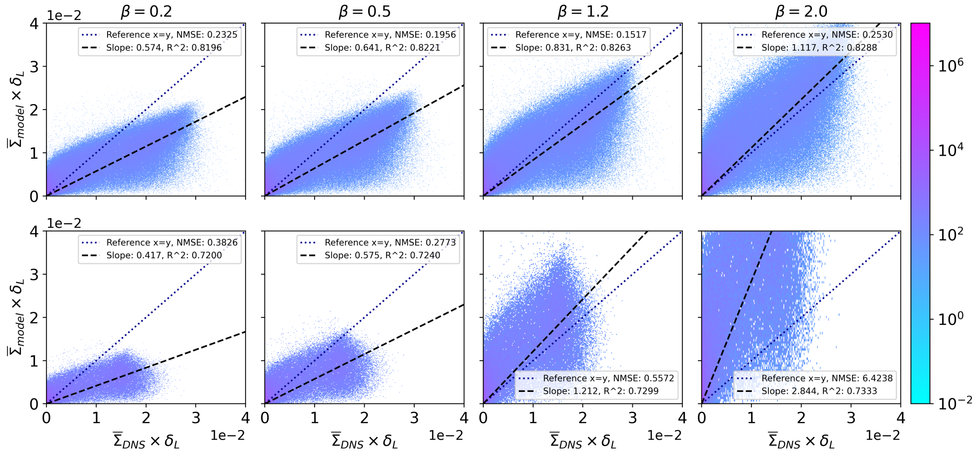

Fig. S1 JPDFs of the filtered flame surface density of the methane/air flame (R4-K1) from the Charlette model with different β values compared against the DNS. The top row of figures are for a filter size of 8 while the bottom row is for a filter size of 16.

2 Simple tests and model validation

Figure S2 (top row) shows the 2D contours of $\overline{\Sigma}$ predicted with three different models compared to the DNS results filtered with a box filter of size 16. For larger filter sizes, the subgrid-scale contributions become more important and therefore, the lack of a subgrid-scale model results in $\overline{\Sigma}$ being significantly underpredicted. The Charlette model shows an improvement in results where the model is able to capture some of the subgrid-scale contributions. However, $\overline{\Sigma}$ is slightly underpredicted in most areas. On the other hand, the CNN model shows excellent performance. Unlike the Charlette model, the CNN model is able to accurately reproduce the $\overline{\Sigma}$ field. Even with larger filter sizes, the CNN model is still able to accurately capture the magnitude and spatial distribution of the $\overline{\Sigma}$ field.

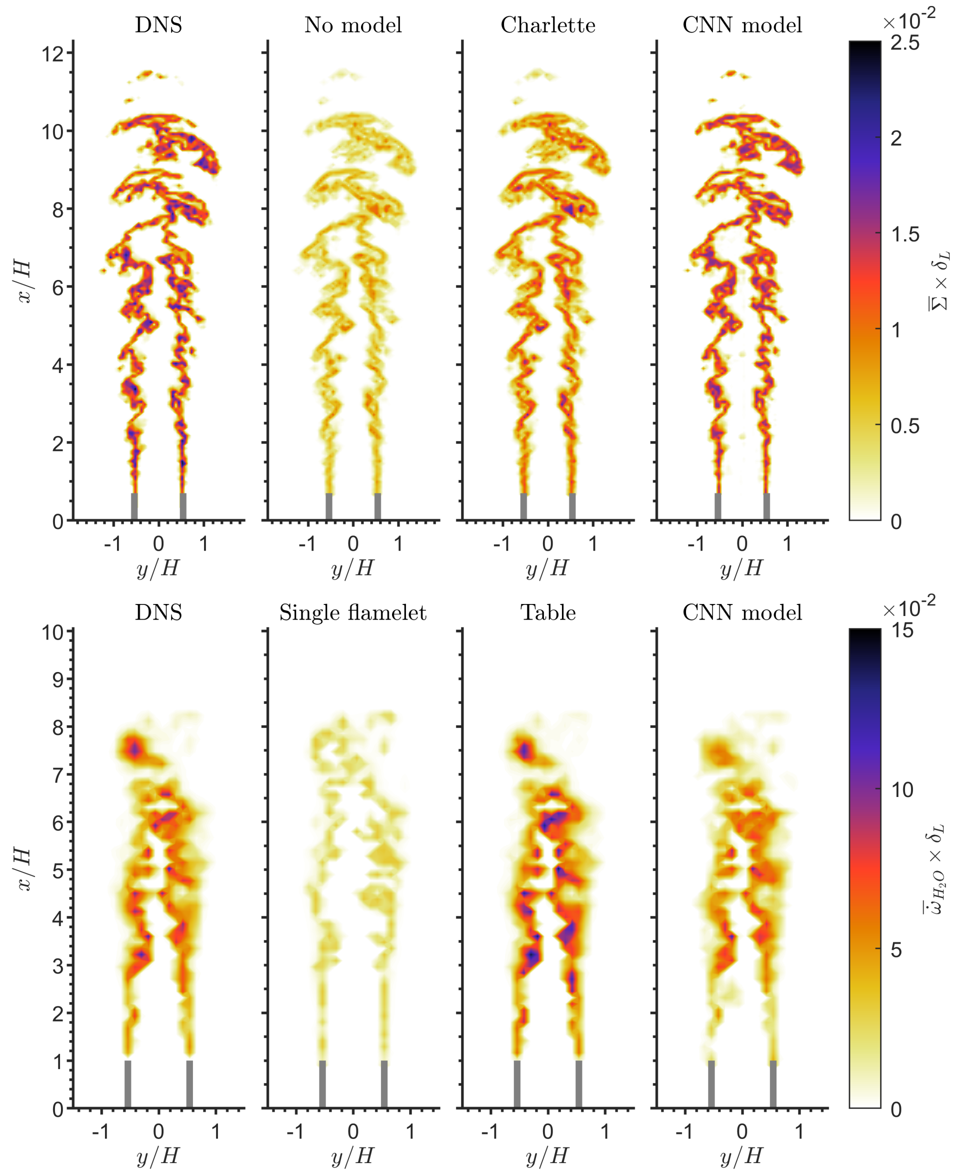

Fig. S2 Top row: 2D contours of the filtered flame surface density of the methane/air flame (R4-K1) from three different models compared against the DNS. Bottom row: 2D contours of the filtered progress variable source term of the hydrogen/air flame from three different models compared against the DNS. Both flames are filtered with a box filter of size 16.

Figure S2 (bottom row) shows the 2D contours of $\overline{\dot{\omega}}_{\mathrm{H_2O}}$ obtained from the CNN model and two additional flamelet-based models compared to $\overline{\dot{\omega}}_{\mathrm{H_2O}}$ obtained from the hydrogen/air DNS for a filter size of 16. The single flamelet model performs poorly as

it significantly underpredicts $\overline{\dot{\omega}}_{\mathrm{H_2O}}$. The model does not capture the enhanced reaction rates that occur in thermodiffusively unstable hydrogen/air flames. In addition, the single flamelet model predicts $\overline{\dot{\omega}}_{\mathrm{H_2O}}$ as constant along the flame front and therefore, cannot capture any local variations. In contrast, the flamelet table, which takes into account the variations of mixture fraction within the flame, significantly improves the prediction accuracy. The model is able to capture the local variations of $\overline{\dot{\omega}}_{\mathrm{H_2O}}$. However, the model slightly overpredicts $\overline{\dot{\omega}}_{\mathrm{H_2O}}$ at certain locations. On the other hand, the CNN model shows excellent performance. Even with larger filter sizes, the CNN model is still able to accurately capture the $\overline{\dot{\omega}}_{\mathrm{H_2O}}$ field, closely matching the DNS results.

For a more detailed comparison, Figure S3 (top row) shows the JPDFs of $\overline{\Sigma}$ between the DNS and the model predictions. The JPDF shows that without a model for the subgrid-scale, $\overline{\Sigma}$ is mostly underpredicted leading to a relatively high NMSE. In comparison, the Charlette model shows a significant improvement, the NMSE is reduced by almost a factor of two and the regression line aligns closer to the equality line, where the predicted value is equal to the DNS value. On the other hand, the CNN model shows a clear improvement in the results compared to the Charlette model. The predictions are in good agreement with the DNS results, where the regression line aligns closely with the equality line and has a low scatter. Figure S3 (bottom row) shows the

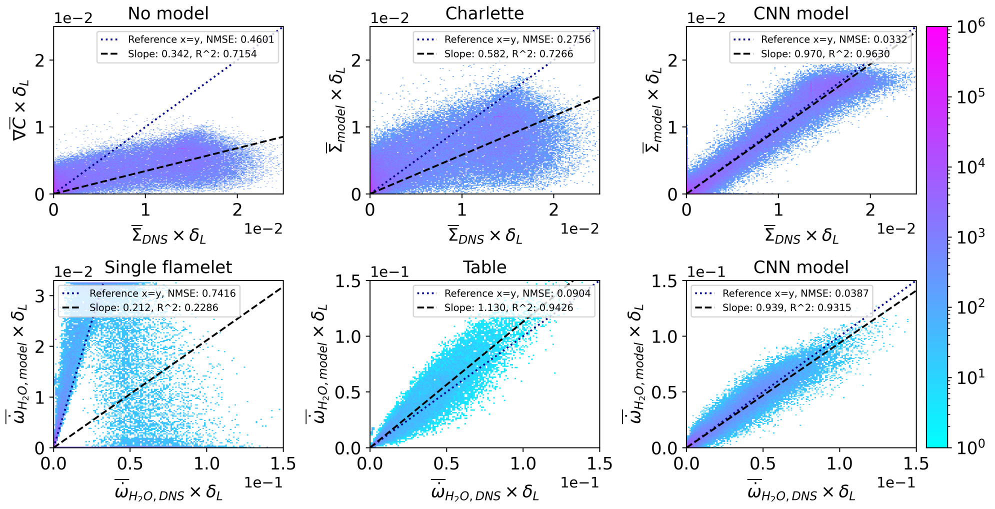

Fig. S3 Top row: JPDFs of the filtered flame surface density of the methane/air flame (R4-K1) from three different models compared against the DNS. Bottom row: JPDFs of the filtered progress variable source term of the hydrogen/air flame from three different models compared against the DNS. Both flames are filtered with a box filter of size 16.

JPDFs of $\overline{\dot{\omega}}_{\mathrm{H_2O}}$ between the DNS and the model predictions. The single-flamelet model shows the worst performance among the evaluated models. It significantly underpredicts $\overline{\dot{\omega}}_{\mathrm{H_2O}}$, which leads to a relatively high NMSE. In addition, the predictions of this model show a significant scatter, leading to a low R^2 value, indicating a poor fit to the

DNS results. In contrast, the flamelet table model shows a significant improvement in prediction accuracy. Its predictions closely align with the DNS results and show only minimal scatter along the regression line. This indicates a strong correlation and higher reliability in capturing the expected behaviour of $\overline{\dot{\omega}}_{\mathrm{H_2O}}$. The performance of the CNN model is very similar to that of the flamelet table. The predictions of the CNN model are very close to the DNS results, with minimal scatter along the regression line. Moreover, even with large filter sizes, the CNN model outperforms the flamelet table by providing slightly more accurate predictions, as evidenced by a lower NMSE.

3 Difference between box and Gaussian filters

Figure S4 shows the JPDFs that highlight the differences between box and Gaussian filters when filtering DNS data. The JPDFs between the filtered progress variable ($\overline{C}$) and the filtered flame surface density (i.e. $\overline{\Sigma} = \overline{|\nabla C|}$) show the difference in filtering between the two filter types. For small filter sizes, both box and Gaussian models behave similarly. The amount of data filtered by both models is very similar. However, with a larger filter size of 16, the Gaussian filter filters more data compared to the box filter, which can be recognised by the slightly lower $\overline{\Sigma}$ values in the JPDF of the Gaussian filter. In addition, the JPDFs of the Gaussian filter have a slightly lower scatter compared to the JPDFs of the box filter. This indicates that the Gaussian filter is able to preserve the locality of the information somewhat better than a corresponding box filter.

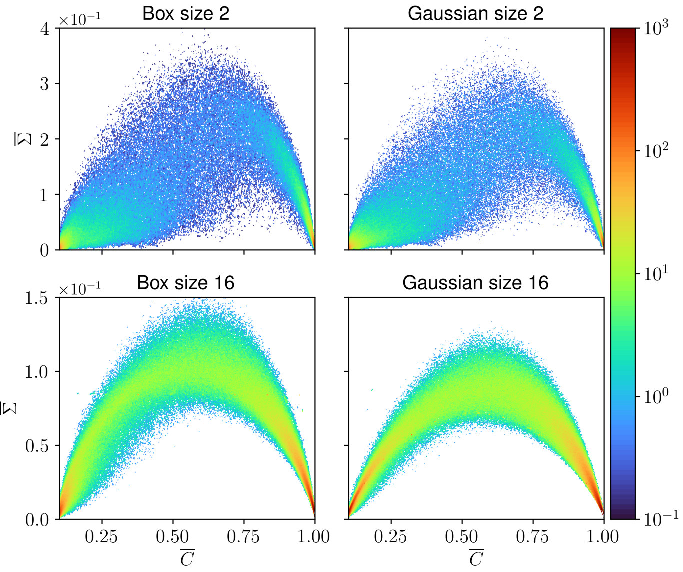

Fig. S4 JPDFs between the filtered progress variable and filtered flame surface density showing the difference between the box and Gaussian filters. The results for the methane/air (R4-K1) flame is shown here.
